# Biomarkers of delirium risk in older adults: a systematic review and meta-analysis

**DOI:** 10.3389/fnagi.2023.1174644

**Published:** 2023-05-12

**Authors:** Lucía Lozano-Vicario, Antonio García-Hermoso, Bernardo Abel Cedeno-Veloz, Joaquín Fernández-Irigoyen, Enrique Santamaría, Román Romero-Ortuno, Fabricio Zambom-Ferraresi, Mikel L. Sáez de Asteasu, Ángel Javier Muñoz-Vázquez, Mikel Izquierdo, Nicolás Martínez-Velilla

**Affiliations:** ^1^Geriatric Unit, Hospital Universitario de Navarra (HUN), Pamplona, Spain; ^2^Navarrabiomed, Hospital Universitario de Navarra (HUN), Universidad Pública de Navarra (UPNA), Instituto de Investigación Sanitaria de Navarra (IdiSNA), Pamplona, Spain; ^3^Proteomics Unit, Navarrabiomed, Hospital Universitario de Navarra (HUN), Universidad Pública de Navarra (UPNA), Instituto de Investigación Sanitaria de Navarra (IDISNA), Pamplona, Spain; ^4^Global Brain Health Institute, Trinity College Dublin, Dublin, Ireland; ^5^CIBER of Frailty and Healthy Aging (CIBERFES), Instituto de Salud Carlos III, Madrid, Spain; ^6^Orthopaedic Surgery and Traumatology Department, Clínica Universidad de Navarra, Pamplona, Spain

**Keywords:** delirium, cognitive impairment, biomarkers, neuroinflammation, older people

## Abstract

Delirium is a neuropsychiatric syndrome associated with increased morbidity and mortality in older patients. The aim of this study was to review predictive biomarkers of delirium in older patients to gain insights into the pathophysiology of this syndrome and provide guidance for future studies. Two authors independently and systematically searched MEDLINE, Embase, Cochrane Library, Web of Science and Scopus databases up to August 2021. A total of 32 studies were included. Only 6 studies were eligible for the meta-analysis, pooled results showed a significant increase in some serum biomarkers (C-reactive protein [CRP], tumour necrosis factor alpha [TNF-α] and interleukin-6 [IL-6]) among patients with delirium (odds ratio = 1.88, 95% CI 1.01 to 1.637; I2 = 76.75%). Although current evidence does not favour the use of any particular biomarker, serum CRP, TNF-*α*, and IL-6 were the most consistent biomarkers of delirium in older patients.

## Introduction

Delirium is a severe neuropsychiatric syndrome characterized by an acute change in attention and other aspects of cognition such as altered arousal, disorientation, psychosis or mood disturbance (Sachdev et al., 2014). Approximately 23% of hospitalized older adults develop delirium in medical settings (Gibb et al., 2020); this proportion increases to 13–50% in noncardiac surgical settings (Inouye et al., 2014), 6–74% in inpatient palliative care units (Watt et al., 2019) and 50–70% among mechanically ventilated patients (Krewulak et al., 2018). Delirium is associated with multiple adverse outcomes, such as higher length of stay, higher risk of iatrogenic and medical complications during hospitalization (falls, malnutrition, dehydration, aspiration pneumonia, immobility, bedsores), increased disability, cognitive impairment, mental illnesses (depression, anxiety, posttraumatic stress disorder) and mortality (Abelha et al., 2013; Martínez-Velilla et al., 2016; Dharmarajan et al., 2017; Altman et al., 2018; Israni et al., 2018; Sillner et al., 2019; Welch et al., 2019; Hayhurst et al., 2020).

The risk of delirium is determined by predisposing factors (for example, pre-existing cognitive impairment, advanced age or frailty) and precipitating factors (acute insults such as surgery, infections or metabolic decompensations) (Wilson et al., 2020). Because multiple factors are implicated in the aetiology of delirium, there are likely several neurobiological processes that contribute to its pathophysiology, that remains unclear. During delirium, a reversal of the relationship between the dorsolateral prefrontal cortex (part of the executive network) (Choi et al., 2012) and the posterior cingulate cortex (involved in the default mode network) (Raichle, 2015) has been observed, contributing to changes in behaviour and attention. In addition, reduced brain efficiency and connectivity strength (especially in subcortical regions related to arousal) have been found in patients with delirium. Delirium can be defined as a failure of a vulnerable brain to show resilience in response to an acute stressor (van Montfort et al., 2019). This vulnerability can be caused by several processes such as impaired brain network connectivity which leads to neurotransmitter disturbance in cholinergic and noradrenergic neurons (Morandi et al., 2012), neuroinflammatory and glial cell changes which causes an exacerbated pro-inflammatory response to noxious insults (Murray et al., 2012) and vasculature dysfunction which produces endothelial injury, blood–brain barrier (BBB) damage and impaired brain perfusion (Cavallari et al., 2016).

Given the complexity of these processes, the use of biomarkers has become widespread for identification of delirium and its risk. The World Health Organization (WHO) defines a biomarker as any substance, structure, or process that can be measured in the body or its products that can influence or predict the incidence of outcome or disease [World Health Organization (WHO), 2001]. Biomarkers are objective and quantifiable characteristics of biological processes, which makes them a reliable and reproducible measure (Strimbu and Tavel, 2010). They are categorized into three patterns: (1) risk markers, which indicate the risk of a particular disease, (2) disease markers, which are correlated with the onset or recovery of a disease, and (3) end-products, which indicate disease resolution (Aronson and Ferner, 2017). The identification of biomarkers associated with delirium may help clarify its pathophysiology and aid in the prediction, diagnosis and management of this syndrome. Although few systematic reviews have been recently carried out on delirium biomarkers, they present important limitations, such as restriction to a single fluid (Hall et al., 2011, 2018; Ida et al., 2020) or a single biomarker (Liu et al., 2018; Narayanan et al., 2021), different types of mixed biomarkers (Chu et al., 2011; Dunne et al., 2021) and the absence of a focus on older adults, in whom delirium risk is the greatest.

Thus, the aim of this study was to systematically review the current literature on delirium biomarkers and identify risk markers in older adults to summarize the existing knowledge, enhance our understanding regarding the pathophysiology and provide methodological guidance for future studies.

## Materials and methods

### Search strategy

This systematic review was undertaken in accordance with the guidelines described by the Preferred Reporting Items for Systematic Reviews and Meta-Analyses (PRISMA) (Liberati et al., 2009). We asked the question whether incident delirium was associated with biomarkers and summarized data extracted from included studies.

Two reviewers (LL and BAC) searched studies published before 17 August 2021 that investigated delirium biomarkers. Searches were performed using a comprehensive text-word and Medical Subject Headings-based electronic search of MEDLINE, EMBASE, The Cochrane Library, Web of Science and Scopus. Primary key words included: “delirium” and “biomarker” used in combination with additional key words as: “cognitive dysfunction,” “acute confusion state,” “acute brain failure,” “postoperative delirium” or “postoperative cognitive disorder.” Boolean operators were used to combine search terms above (Supplementary material). Authors with specialist knowledge of the subject extracted the data. To avoid bias, results were based on analysis by LL, AC, NM, and AG. Variations in interpretation were reviewed, and any disputes were resolved by NM. The review protocol was registered in PROSPERO (CRD42021281272).

### Selection criteria

The inclusion criteria were:

– Study design: case–control, cohort study or case series with non-delirious subjects as controls
– Language of publication: English or Spanish
– Year of publication: all studies until 17.08.2021
– Study subjects: mean patient aged ≥65 years
– Diagnostic criteria of delirium:
  ο Diagnostic Statistical Manual of Mental Disorders (DSM)
  ο International Classification of Diseases (ICD)
  ο Delirium assessment tool based on DSM or ICD
– Source of biomarker: cerebrospinal fluid, blood or other body fluids.
– Measurement methods: methods that provide quantitative (i.e., ELISA, RIA) or detailed qualitative data (i.e., proteomics)
– Full text available (detailed information)

The exclusion criteria were:

– Reviews, case reports or comments, letters, personal opinions, book chapters and conference abstracts
– Randomized controlled trials (RCTs) measuring the effects of drugs on delirium incidence
– Studies where biomarkers were not identified
– Association between biomarkers and delirium in experimental studies (*in vitro* or *in vivo* animal studies)
– No identifiable delirium/no delirium subgroups
– Delirium of other specific causes (delirium tremens or other alcohol withdrawal states, Wernicke’s encephalopathy, neuropsychiatric systematic lupus erythematosus).

### Data extraction and synthesis

Two authors independently screened the remaining literature for useable data and extracted it, and this was checked by another reviewer. Articles that met the inclusion criteria were reviewed and we recorded (if available) the following information: (1) Author and year of publication, (2) study design and setting (i.e., orthopedics, cardiac surgery, ICU patients…), (3) number and characteristics of patients included (age, sex, delirious/no delirious), (4) method used to diagnose delirium and delirium severity, (5) presence of cognitive impairment and method of assessment, (6) type of biomarker analyzed and levels, (7) method of obtaining the samples, (8) analytical laboratory methodology, and (9) main study findings. In cases in which more than one publication of the same trial existed, only the latest publication with the most complete data was included. Disagreements were settled by consensus.

In the case of studies that included both people younger and older than 65 years and specific data for older adults were included in the publication, we only used data from individual participants ≥65 years. If individual participant data for the subgroup of interest could not be obtained, we included studies if greater than or equal to 80% of the participants were ≥ 65 years.

### Endpoints

The main risk markers of delirium are shown in Table 1.

**Table 1:** Risk markers of delirium.

Type of biomarker	Biomarkers analyzed	Number of studies	Authors
Neurotransmitters	AChE, BChE, Ach, Kyneurine/Tryptophan, IDO, HVA	5	Adam et al. (2020), Cerejeira et al. (2012), de Jonghe et al. (2012), Ma et al. (2020), Osse et al. (2012)
Hormones	Estradiol, cortisol, leptin	3	Avila-Funes et al. (2015), Li et al. (2017), Ma et al. (2020)
Biomarkers of neuronal damage	S100B, NfL, pNfLH, UCHL-1, neurogranin	5	Fong et al. (2020), Halaas et al. (2021), Hov et al. (2017), Saller et al. (2019), Szwed et al. (2020)
Biomarkers of neuroinflammation	IFN-γ, IFN-*α*2, IGF-1, GFAP, CRP, hsCRP, CAR, TNF-*α*, IL-1ra, IL-1ß, IL-2, IL-4, IL-5, IL-6, IL-8, IL-10, IL-12p70, MCP-1, MIP-1*α*, MIP-1β, RAGE, calprotectin, MRP8/14, CHI3L1, neopterin	17	Cape et al. (2014), Cerejeira et al. (2012), Chen et al. (2020), Chen et al. (2019), Chu et al. (2016), Çinar et al. (2014), Dillon et al. (2017), Fong et al. (2020), Hirsch et al. (2016), Katsumi et al. (2020), Kaźmierski et al. (2021), Osse et al. (2012), Peng et al. (2019), Sajjad et al. (2020), Saller et al. (2019), Shen et al. (2016), Szwed et al. (2020)
Biomarkers of dementia	t-tau, p-tau, tau, Aß40, Aß42	5	Fong et al. (2020), Hirsch et al. (2016), Hov et al. (2017), Pan et al. (2019), Saller et al. (2019)
Genetics	miR-210	1	Chen et al. (2020)
Metabolomics, lipidomics and proteomics	PE (40:7e), PE (40:6), PE (38:7e), PC (40:6), PC (33:1), Cer-NS, SM VSTM2B, FA5, Spermidine, Glutamine, Putrescine, AZGP1, CHI3L1/YKL-40	5	Han et al. (2020a,b), Pan et al. (2019), Vasunilashorn et al. (2019), Vasunilashorn et al. (2021)
Others	Creatinine, PLR and PWR, NSP, VILIP-1, BDNF	4	Bakker et al. (2012), Kotfis et al. (2019), Szwed et al. (2020), Wyrobek et al. (2017)

AChE, acetylcholinesterase; BChE, butyrylcholinesterase; Ach, acetylcholine; IDO, indoleamine 2,3-dioxygenase; HVA, homovanillic acid; IFN, human interferon; IGF-I, insulin-like growth factor-1; CRP=C-reactive protein; CAR, C-reactive protein-to-albumin ratio; hsCRP, high-sensitivity C-reactive protein; IL, interleukin; TNF, tumor necrosis factor; S100(B), calcium-binding protein (B); GFAP, glial fibrillary acidic protein; MCP-1, monocyte chemoattractant protein 1; NfL, neurofilament light; pNfLH, phosphorylated axonal neurofilament subunit H; NSE, neuron specific enolase; Aß, amyloid-beta; t-tau, total tau; p-tau, phosphorylated tau; miRNAs, MicroRNAs; UCHL-1, ubiquitin carboxyl-terminal hydrolase L1; PE, phosphatidylethanolamine; PC, phosphatidylcholine; SM, sphingomyelin; Cer-NS, ceramide non-hydroxyfatty acid-sphingosine; VSTM2B, transmembrane domain-containing protein 2B; FA5, coagulation factor V; MIP, macrophage inflammatory protein; RAGE, Receptor for advanced glycation end products; WDC, white blood cell count; PWR, lower platelet-to-white blood cell ratio; PLR, lower platelet-to-lymphocyte ratio; CHI3L1, Chitinase 3-Like 1 glycoprotein (also known as YKL-40 [tyrosine (Y), lysine (K), leucine (L)] with molecular weight of 40); NSP, neuroserpin; VILIP-1, visinin-like protein-1; AZGP1, zinc alpha-2 glycoprotein; MRP, Macrophage inflammatory protein; BDNF=Brain-derived neurotrophic factor.

### Assessment of study quality

Study quality and risk of bias were assessed independently by two of the authors using the Newcastle-Ottawa scale (NOS), which is designed for assessing common causes of bias in cohort studies. The NOS score ranges from 0 to 9 stars. A quality score was calculated based on three major components: (1) the selection of study groups (0–4 stars), (2) the comparability of study groups (0–2 stars), and (3) ascertainment of the exposure and outcome of interest. Overall, the quality of studies was deemed as poor (0 to 3 stars), fair (4 to 6 stars) or excellent (7 to 9 stars). Disagreement was resolved by discussion and consensus. The summary of assessment of risk of bias and additional comments on study quality are shown in Supplementary material.

### Statistical analyses

All analyses were conducted using the DerSimonian-Laird random-effects inverse variance model using STATA software (version 17; StataCorp, College Station, TX, United States). Data were pooled only if biomarkers were reported in at least two studies. We used the odds ratios (OR) as the main effect size for the present study. We converted other estimations (e.g., standardized regression coefficients, standardized mean differences) to OR according to their corresponding formulas. A subgroup analysis according to inflammatory parameters was also included for CRP, TNF-*α*, and IL-6 data. Heterogeneity across studies was calculated using the inconsistency index (*I*^2^) (Cooper et al., 1994), and Egger’s regression intercept test was used to detect small-study effects bias (Egger et al., 1997; Higgins et al., 2003). No other sub-group analysis was performed due to the limited number of studies.

## Results

### Results of the literature search

**Figure 1:**
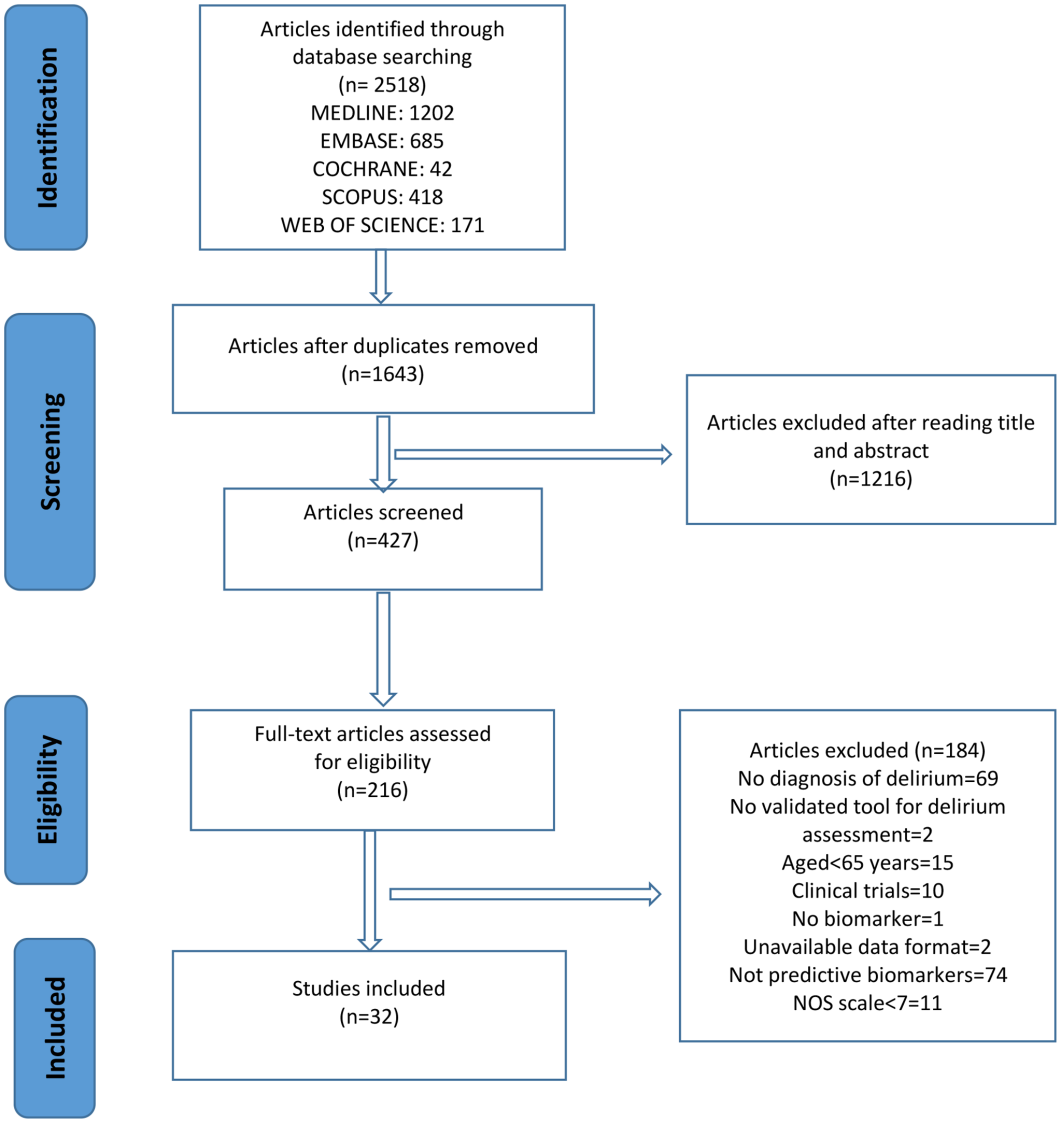
Flowchart.

We located 2,518 records, of which 1,644 remained after removing duplicates. After screening the titles and abstracts, 217 studies remained for full text review. Ultimately, 32 articles were included (shown in Figure 1). The major characteristics of the included studies are presented in Supplementary material, and a summary of the main results is shown in Table 2.

**Table 2:** Summary of main results of risk markers of delirium.

Biomarker	Type of biomarker	Number of studies	Results
AChE	Neurotransmitter	2	↓
BChE	Neurotransmitter	2	In one study ↓ but in the other study no association was found
Ach	Neurotransmitter	1	↓
Kynurenine/Tryptophan	Neurotransmitter	1	↑
IDO	Neurotransmitter	1	↑
HVA	Neurotransmitter	1	↑
Estradiol	Hormone	1	↑
Cortisol	Hormone	2	In one study ↑ but in the other study no association was found
Leptin	Hormone	1	↓
S100B	Neuronal damage	1	↑
NfL and pNfLH	Neuronal damage	3	In one study ↑ predicted POD and in 2 studies no association was found
UCHL-1	Neuronal damage	1	No association
Neurogranin	Neuronal damage	1	No association
IFN-γ	Neuroinflammation	2	In one study ↓ and in the other study, no association was found
IFN-*α*2	Neuroinflammation	1	↓
IGF-1	Neuroinflammation	4	In 2 studies ↓ and in other 2 studies no association was found
GFAP	Neuroinflammation	4	No association
CRP	Neuroinflammation	7	In 5 studies ↑ predicted delirium but in other 2 studies no association was found
hsCRP	Neuroinflammation	1	↑
CAR	Neuroinflammation	1	↑
TNF-*α*	Neuroinflammation	6	In 1 study ↑ predicted delirium but in other 5 studies no association was found
IL-1ra	Neuroinflammation	1	No association
IL-1ß	Neuroinflammation	3	In 1 study ↑ predicted delirium but in other 2 studies no association was found
IL-2	Neuroinflammation	1	No association
IL-4	Neuroinflammation	1	↓
IL-5	Neuroinflammation	1	↓
IL-6	Neuroinflammation	7	In 4 studies ↑ predicted delirium but in other 3 studies no association was found
IL-8	Neuroinflammation	3	In 2 studies ↑ predicted delirium but in 1 study, no association was found
IL-10	Neuroinflammation	2	No association
IL-12p70	Neuroinflammation	1	↓
MCP-1	Neuroinflammation	2	In 1 study ↑ predicted delirium but in other study no association was found
MIP-*1α*	Neuroinflammation	1	↑
MIP-1*ß*	Neuroinflammation	1	↑
RAGE	Neuroinflammation	1	↓
Calprotectin	Neuroinflammation	1	↑
MRP8/14	Neuroinflammation	1	No association
CHI3L1	Neuroinflammation	1	No association
Neopterin	Neuroinflammation	1	↑
t-tau	Dementia	2	No association
p-tau	Dementia	2	In 1 study ↑ but in other study no association was found
tau	Dementia	1	↑
A*ß*40	Dementia	1	↓
A*ß*42	Dementia	2	↓
miR-210	Genetics	1	↑
PE (40:7e)	Lipidomics	1	↓
PE (40:6)	Lipidomics	1	↓
PE (38:7e)	Lipidomics	1	↓
PC (40:6)	Lipidomics	1	↓
PC (33:1)	Lipidomics	1	↓
Cer-NS	Lipidomics	1	↑
SM	Lipidomics	1	↑
VSTM2B	Proteomics	1	↓
FA5	Proteomics	1	↓
Spermidine	Metabolomics	1	↑
Glutamine	Metabolomics	1	↑
Putrescine	Metabolomics	1	↑
AZGP1	Proteomics	1	↓
CHI3L1/YKL-40	Proteomics	1	↑
Creatinine	Other	1	↑
PLR and PWR	Other	1	↓
NSP	Other	1	↑
VILIP-1	Other	1	No association
BDNF	Other	1	↓

↓ = low levels of this biomarker were associated with delirium.

↑ = high levels of this biomarker were associated with delirium.

### Study population

There was diversity between the clinical settings of the studies included in this systematic review. Most of them (30 studies) were carried out in surgical patients: 10 studies in cardiac surgery patients, 14 in orthopaedic surgery patients, 2 in cancer surgery and 4 in other surgery patients. Only 2 studies were conducted among medical inpatients (1 in the hospital ward and the other in the ICU).

The age of the patients included in the studies varied between 67 and 88 years. Seventeen studies included more males than females, while females were more prevalent in 15 studies.

### Delirium assessment, delirium severity and delirium subtype

The Confusion Assessment Method was the most commonly used tool for assessing delirium (Inouye et al., 1990; Helfand et al., 2021), followed by the Confusion Assessment Method for Intensive Care Unit (CAM-ICU) (Gusmao-Flores et al., 2012), DSM and Delirium Rating Scale (DRS) (Trzepacz, 1999). Eighteen studies applied the CAM to define delirium, nine studies used the CAM-ICU, three studies assessed delirium with the DSM criteria, one study used the DRS and DSM criteria and one study used both the CAM and the CAM-ICU.

The severity of delirium was assessed in ten studies. The most commonly used tool was the Memorial Delirium Assessment Scale (MDAS), followed by the Confusion Assessment Method-Severity (CAM-S) and the DRS.

The subtype of delirium was only evaluated in one study, which used the Richmond Agitation-Sedation Scale (RASS).

### Sample characteristics

Most of the studies collected blood samples. Cerebrospinal fluid (CSF) was collected in 4 studies and both blood and CSF were collected in 5 studies.

### Risk markers of delirium

#### Neurotransmitters

Serum anticholinergic activity (SAA) was investigated as a risk marker of delirium in three studies. There were mixed results, as 2 studies (Cerejeira et al., 2012; Adam et al., 2020) demonstrated that lower preoperative acetylcholinesterase (AChE) activity was related to postoperative delirium (POD). One study showed that lower preoperative butyrylcholinesterase (BChE) activity was correlated with POD (Cerejeira et al., 2012), but another study did not find an association (Adam et al., 2020). Low levels of acetylcholine (Ach) before surgery were an independent risk factor for POD (Ma et al., 2020). de Jonghe et al. (2012) found that a higher preoperative kynurenine/tryptophan ratio and higher indoleamine 2,3-dioxygenase (IDO) activity were risk markers for POD. Higher postoperative levels of homovanillic acid (HVA) were associated with POD (Osse et al., 2012).

#### Hormones

Three papers explored a link between serum hormones and incident delirium. One study found that medical inpatients with higher levels of estradiol had an increased risk of developing delirium (Avila-Funes et al., 2015). Two studies investigated cortisol; Ma et al. (2020) found that higher preoperative cortisol levels were an independent risk factor for delirium, but Avila-Funes et al. (2015) did not find any associations between cortisol in medical inpatients and incident delirium. Li et al. (2017) found that low leptin levels at ICU admission were independently associated with delirium.

#### Neuronal damage biomarkers

Five studies focused on the relationship between biomarkers of neuronal damage and delirium. Hov et al. (2017) found that higher preoperative levels of S100 calcium-binding protein B (S100B) in CSF correlated with POD in patients who also had pathological levels of p-tau. Serum Neurofilament light (NfL) was assessed in two studies. Fong et al. (2020) found that higher preoperative levels were associated with POD and POD severity, but Saller et al. (2019) did not find differences. Szwed et al. (2020) evaluated serum phosphorylated axonal neurofilament subunit H (pNfLH), but no association with delirium was found. One study investigated serum ubiquitin carboxyl-terminal hydrolase L1 (UCHL-1) (Fong et al., 2020) and another study examined CSF neurogranin (Halaas et al., 2021), but no differences were reported between these biomarkers and delirium.

#### Neuroinflammatory markers

Nineteen studies analysed the role of biomarkers of neuroinflammation in delirium. The most investigated biomarkers in this field were C-reactive protein (CRP), tumour necrosis factor (TNF) and interleukin-6 (IL-6). Seven studies explored CRP. Five studies found that higher preoperative CRP in serum correlated with POD (Cerejeira et al., 2012; Shen et al., 2016; Dillon et al., 2017; Vasunilashorn et al., 2019; Chen et al., 2020); however, two studies did not find differences (Çinar et al., 2014; Katsumi et al., 2020). Kaźmierski et al. (2021) found that high levels of high-sensitivity C-reactive protein (hsCRP) in serum were also associated with POD, and Peng et al. (2019) also analysed the C-reactive protein-to-albumin ratio (CAR), showing that higher preoperative levels of serum CAR were an independent risk factor for POD. TNF-*α* was analysed in six studies; only one found that higher preoperative levels of TNF-*α* were related to POD but not significantly (Peng et al., 2019); in the rest of the studies, no differences were found (Cerejeira et al., 2012; Çinar et al., 2014; Hirsch et al., 2016; Chen et al., 2020; Sajjad et al., 2020). Seven studies investigated IL-6: four studies found that higher preoperative levels of IL-6 were related to delirium (Hirsch et al., 2016; Shen et al., 2016; Chen et al., 2019; Peng et al., 2019), while three studies found no differences (Cerejeira et al., 2012; Chen et al., 2020; Katsumi et al., 2020).

Other interleukins were explored, such as IL-1ra (Cape et al., 2014), IL-2 (Hirsch et al., 2016) and IL-10 (Hirsch et al., 2016), but they were not found to be related to the incidence of delirium. Cape et al. (2014) found that higher IL-1ß in CSF correlated with POD, but in two other studies, no association was found (Cerejeira et al., 2012; Sajjad et al., 2020). Hirsch et al. (2016) also showed that lower levels of IL-4, IL-5 and IL-12p70 were related to POD. IL-8 was analysed in three studies; in two of them, higher preoperative levels in CSF correlated with POD (Hirsch et al., 2016; Sajjad et al., 2020), but in one study, no differences were found (Cerejeira et al., 2012). Monocyte chemoattractant protein 1 (MCP-1) was analysed in two studies; one of them found that higher serum levels before surgery were associated with delirium (Kaźmierski et al., 2021), but the other one did not find any difference (Hirsch et al., 2016). Hirsch et al. (2016) also studied macrophage inflammatory protein (MIP), macrophage inflammatory protein (MRP), receptor for advanced glycation end products (RAGE) and calprotectin and found that POD was related to higher preoperative levels of MIP-1*α* MIP-1ß and calprotectin but not RAGE or MRP8/14. Preoperative chitinase 3-like 1 glycoprotein (CHI3L1) was evaluated in one study, but no association was found with delirium (Katsumi et al., 2020).

Human interferon-γ (IFN-γ) was investigated in two studies; one study found that lower preoperative IFN-γ in plasma was related to POD (Hirsch et al., 2016), but the other one did not find any differences in CSF and blood (Cape et al., 2014). Hirsch et al., 2016 also found that lower preoperative human interferon-*α*2 (IFN-*α*2) in CSF was implicated in the development of POD. Four studies explored insulin-like growth factor-1 (IGF-1). Two studies found that lower preoperative levels of IGF-1 were associated with POD (Çinar et al., 2014; Shen et al., 2016), but the other two studies did not find any differences (Cape et al., 2014; Chu et al., 2016). Glial fibrillary acidic protein (GFAP) was also investigated in 4 studies, but no association was found in any of them (Cape et al., 2014; Saller et al., 2019; Fong et al., 2020; Szwed et al., 2020). Only IL-6, TNF-*α* and CRP could be examined *via* meta-analysis, as each biomarker was investigated in 6 or more studies.

**Figure 2:**
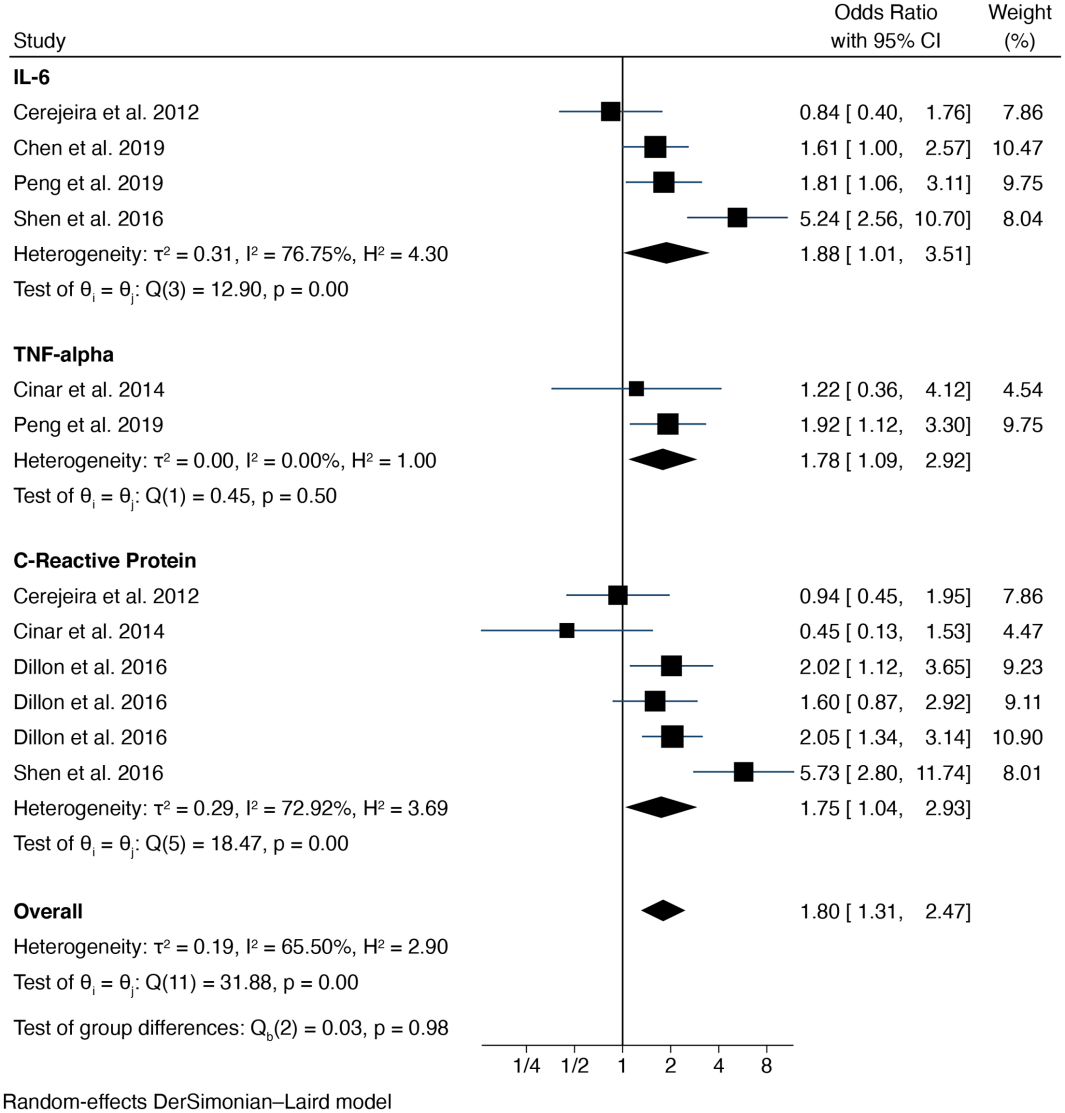
Forest plot.

The major findings of this meta-analysis are presented in Figure 2. Overall, pooled analysis showed a significant increase in some serum biomarkers (i.e., CRP, TNF-*α*, and IL-6) of patients who developed delirium (OR = 1.88, 95% CI 1.01 to 1.637; I^2^ = 76.75%). Egger’s test indicated no small-study effects bias for pooled analysis (*p* = 0.178). Four studies could be included in the meta-analysis of IL-6; the results showed a significant increase in this biomarker in serum (OR = 1.88, 95% CI 1.01 to 1.637) in patients who developed delirium, with high heterogeneity (*τ*^2^ = 0.31, *I*^2^ = 76.75%). Four studies were included in the meta-analysis of CRP, but Dillon et al. (2017) evaluated three different cohorts with three independent analyses, so we included all of them in this meta-analysis. Serum preoperative CRP was significantly high in patients with later POD (OR = 1.75; 95% CI 1.04 to 2.93), with high heterogeneity (*τ*^2^ = 0.29, *I*^2^ = 72.92%).

#### Biomarkers of dementia

Five studies investigated the relationship between biomarkers of dementia and delirium. Two studies analysed total tau (t-tau), but no differences were found between this biomarker and delirium in either blood (Fong et al., 2020) or CSF (Pan et al., 2019). Two studies evaluated phosphorylated tau (p-tau); in one study, higher CSF p-tau correlated with POD (Hov et al., 2017), but in another study, no differences were found between CSF p-tau and delirium (Pan et al., 2019). However, Saller et al. (2019) found that higher preoperative tau in serum correlated with POD. Hirsch et al. (2016) showed that lower preoperative Aβ40 in plasma predicted POD. Aß42 was investigated in two studies; one found that lower preoperative Aß42 in plasma correlated with incident POD (Hirsch et al., 2016), and the other found that lower preoperative Aß42 in CSF predicted POD (Pan et al., 2019).

#### Genetics

Only one study evaluated the implication of genetics in the development of delirium (Chen et al., 2020) showing that higher preoperative expression of miR-210 in blood was a predictor of POD.

#### Metabolomics, lipidomics and proteomics

Five studies explored the role of -omics approaches in delirium. Han et al., showed that CSF lipidomics and metabolomics pointed out that phosphatidylethanolamine (PE), phosphatydilcholine (PC), sphingomyelin (SM) and ceramide non-hydroxyfatty acid-sphingosine (Cer-NS) were related with delirium (Han et al., 2020b). In this study, lower preoperative levels of PE (40:7e), PE (40:6), PE (38:7e), PC (40:6) and PC (33:1) but higher preoperative levels of CER-NS and SM were associated with incident POD. A CSF proteomic study found that lower preoperative levels of transmembrane domain-containing protein 2B (VSTM2B) and coagulation factor V (FA5) were positively correlated with delirium severity (Han et al., 2020a). Vasunilashorn et al., showed that lower preoperative serum levels of zinc alpha-2 glycoprotein (AZGP1) were associated with POD (Vasunilashorn et al., 2019). Moreover, a serum proteomic study found that higher preoperative chitinase 3-like 1 glycoprotein (CHI3LI/KYL-40) were associated with POD (Vasunilashorn et al., 2021). Pan et al., showed that higher preoperative levels of spermidine, glutamine and putrescine in CSF were related with incident delirium (Pan et al., 2019).

#### Others

Bakker et al., showed that higher levels of serum creatinine prior to surgery were an independent predictor of POD (Bakker et al., 2012). Kotfis et al., found that lower preoperative levels of platelet-to-white blood cell ratio (PWR) and lower platelet-to-lymphocyte ratio (PLR) were associated with POD in serum (Kotfis et al., 2019). Szwed et al., investigated the relationship between neuroserpin (NSP) or visinin-like protein-1 (VILIP-1) and delirium (Szwed et al., 2020) in serum. Higher end of surgery to baseline ratio of NSP predicted the occurrence of POD but no differences were found between VILIP-1 and POD. Wyrobeck et al., showed that lower levels of serum Brain-derived neurotrophic factor (BDNF) during surgery were associated with delirium (Wyrobek et al., 2017).

## Discussion

To the best of our knowledge, this is the first review that focuses on predictive biomarkers of delirium in older patients, considering all clinical settings and all types of biological fluids with high-quality studies. The multi-etiological nature of delirium is likely to be reflected in the wide range of biomarkers identified by our study.

### Ach

The neurotransmitter hypothesis suggests that disturbances in neurotransmitter pathways can lead to delirium (Maldonado, 2013). Although precursors of serotonin (tryptophan, phenylalanine and tyrosine), dopamine and noradrenaline may be implicated in the development of delirium, anticholinergic deficiency has been directly related to its pathophysiology (Wang and Shen, 2018). Ach is involved in sleep regulation, cognition and attention. It is well known that an impairment in cholinergic activity is associated with cognitive and attentional changes; current literature also suggests that an impairment in cholinergic activity is associated with delirium (Pasina et al., 2019; Vondeling et al., 2020; Oudewortel et al., 2021; Lisibach et al., 2022). In addition, anticholinergic effects are common among several drugs, and a previous study found that increased anticholinergic burden increases delirium risk (Egberts et al., 2021).

### Cortisol

A disruption of the hypothalamic–pituitary-axis with an increase in cortisol levels has been related to a higher risk of dementia (Ouanes and Popp, 2019) and delirium, particularly in critically ill patients (Mattar et al., 2012; Michels et al., 2019), but evidence is not conclusive. Other hormones, such as estradiol or leptin, may have a role in delirium pathophysiology, but additional research is needed to confirm this hypothesis.

### S100B and NfL

Markers of neuronal damage, such as S100B and NfL (related to disturbances in astrocytic integrity), have been associated with delirium (Mietani et al., 2019, 2021), but these markers of brain injury could be associated with established, and not necessarily incident, delirium (Khan et al., 2011). However, higher levels have been associated with higher severity and worse prognosis of delirium (Gao et al., 2021; Narayanan et al., 2021; Page et al., 2022).

### Tau and Aß

It is well known that cognitive impairment is a clinical risk factor for delirium (Fong et al., 2015, 2017, 2019; Racine et al., 2017). Nevertheless, few studies have been carried out in this field, pointing out that typical markers of dementia, such as tau or Aβ, are associated with delirium development (Wang et al., 2021, 2022).

### Genetics

Another pathway that yields promising results is genetic research in delirium (Van Munster et al., 2009). Although studies in this field are scarce, genetic studies could be interesting translational tools for elucidating the pathophysiology of this syndrome. In addition, genetics are not influenced by the causal combination of predisposing and precipitating factors that affects delirium, so they can offer a more reliable value compared to other biomarkers (Sepulveda et al., 2021).

### Proteomics and metabolomics

Research in proteomics and metabolomics aims to provide a protein or metabolic profile to contribute to delirium diagnosis and prevention (Sfera et al., 2015). There are few and heterogeneous studies in this field due to the technological difficulty associated with mapping the whole molecular landscape of biofluids. However, omics technologies are complementary to the genetic toolbox, detecting, identifying and quantifying alternative molecular biomarkers necessary to understand the dynamics and interactions that occur during delirium development.

Although acetylcholine deficiency, hormonal influence, markers of dementia and biochemical changes observed through proteomics and metabolomics may play an important role in predicting delirium, neuroinflammation theory seems to be the most relevant pathway, at least in the population of this review (most of the studies were carried out in older surgical populations). These findings are consistent with previous reviews that found an association between delirium and neuroinflammation biomarkers (Hall et al., 2018; Liu et al., 2018; Adamis et al., 2021; Dunne et al., 2021; Noah et al., 2021). This hypothesis suggests that peripheral inflammation due to surgery, trauma or infection leads to the activation of the proinflammatory cascade and suppression of anti-inflammatory markers. These stimuli trigger tissue macrophage and blood monocyte activation and secretion of inflammatory mediators such as IL-1, IL-1ß, IL-6, TNF-*α* and prostaglandin E_2_ (PGE_2_). These proinflammatory molecules penetrate the BBB, producing cerebral injury through the activation of microglia that causes brain dysfunction and delirium (Wilson et al., 2020). Interestingly, we conducted a meta-analysis with IL-6, TNF-*α*, and CRP, which were the most investigated biomarkers, and we found that they were statistically significant predictors of delirium.

#### Il-6

IL-6 is a cytokine involved in the immune response and has a notorious role in adult neurogenesis, the process of creating new neurons and glial cells from neural stem cells (oligodendrogliogenesis and astrogliogenesis) in the central nervous system (CNS) (Kang et al., 2020). IL-6 expression is involved in the synthesis of beta-amyloid precursor protein being altered in the brains of Alzheimer’s disease (AD) patients and it is also upregulated whenever neuroinflammation is expected, such as infection or injury (Erta et al., 2012). Noah et al. (2021) found that higher preoperative IL-6 was associated with postoperative delirium. Another meta-analysis performed among surgical patients also found this association (Liu et al., 2018; Adamis et al., 2021). Dunne et al. (2021) showed that early manifestation of systemic inflammation with elevated levels of IL-6 leads to the onset of delirium. However, Hall et al. (2018) did not find a significant correlation between IL-6 measured in CSF and delirium.

#### TNF-*α*

TNF-*α* is a proinflammatory cytokine (Idriss and Naismith, 2000) that has been associated with more rapid cognitive decline in patients with AD (Holmes et al., 2009). Analysis of the same cohort showed that elevated systemic TNF-*α* was associated with an increase in psychobehavioural alterations such as apathy, anxiety, depression and agitation, suggesting that increased systemic TNF-*α* may also have a role in hippocampal neurodegeneration (Holmes et al., 2011). The association of TNF-*α* and delirium is unclear. While some studies showed a positive correlation between TNF-*α* and delirium and our study revealed a significant association, previous meta-analyses by Liu et al. (2018) and Noah et al. (2021) found that preoperative TNF-*α* was significantly higher in the POD group in univariate analysis but not in multivariate analysis.

#### CRP

CRP is a pentameric protein whose circulating concentrations rise in response to inflammation (Pathak and Agrawal, 2019). It is an acute-phase protein of hepatic origin that increases following IL-6 secretion (Sproston and Ashworth, 2018). In the same way that IL-6 is altered in patients with cognitive impairment, a recent review found that CRP is also involved in this mechanism (Qiao et al., 2022). Moreover, several meta-analyses have shown that higher preoperative serum CRP levels are significantly associated with later POD (Liu et al., 2018; Adamis et al., 2021; Dunne et al., 2021; Noah et al., 2021).

### Strengths and limitations

One of the main limitations of the studies included in this systematic review and meta-analysis is their small sample size and the lack of consistent terminology about delirium which has negatively affected the research (different terms such as organic brain syndrome, encephalopathy or acute brain failure can be misleading). Another important drawback is the lack of diversity in patient populations. Most of the studies were carried out in surgical populations, so their results may not be extrapolated to other clinical scenarios (medical inpatients, critically ill patients, etc.). On the other hand, there was little information about geriatric syndromes (frailty, malnutrition, polypharmacy) that may be directly involved not only in the development of delirium but also in the delirium characteristics (severity, subtype, duration, etc.). In addition, it is important to note that the methodological heterogeneity observed across studies might have an impact on the overall outcomes; therefore, the information should be considered with caution when drawing conclusions.

Importantly, this study provides several strengths. Firstly, there was no restriction by year of publication, and only high-quality studies (7 to 9 stars in NOS) with formal delirium diagnosis (DSM criteria, ICD criteria or a tool based on these criteria) with a lower risk of bias were included. Secondly, only studies on risk markers for delirium were included, excluding studies on disease markers or end-products of delirium. Thirdly, we also compared the preoperative state of patients who had not yet developed delirium in the studies that had more than one sample collected at different time points, focusing on predictive biomarkers of delirium and minimizing the risk of bias.

### Challenges and implications for future research

These findings could contribute to the implementation of the International Drive to Illuminate Delirium (IDID) initiative (Khachaturian et al., 2020), providing a new prevention strategy for delirium and, subsequently, helping to preserve cognitive function. Although biomarkers could be an instrument to predict delirium, there are some unanswered questions. Blood-based biomarkers are little invasive, fast and reproducible, giving a quantitative value, and could be easy to measure in routine analysis (accessible in hands of surgeons, primary care physicians or other clinics). However, the validation of these biomarkers in terms of accuracy, sensitivity and specificity remains unknown because many of these inflammatory mediators are elevated in patients with sepsis, trauma or surgery and not only in those who develop delirium. In addition, their affordability and cost-efficiency are also unexplored.

**Figure 3:**
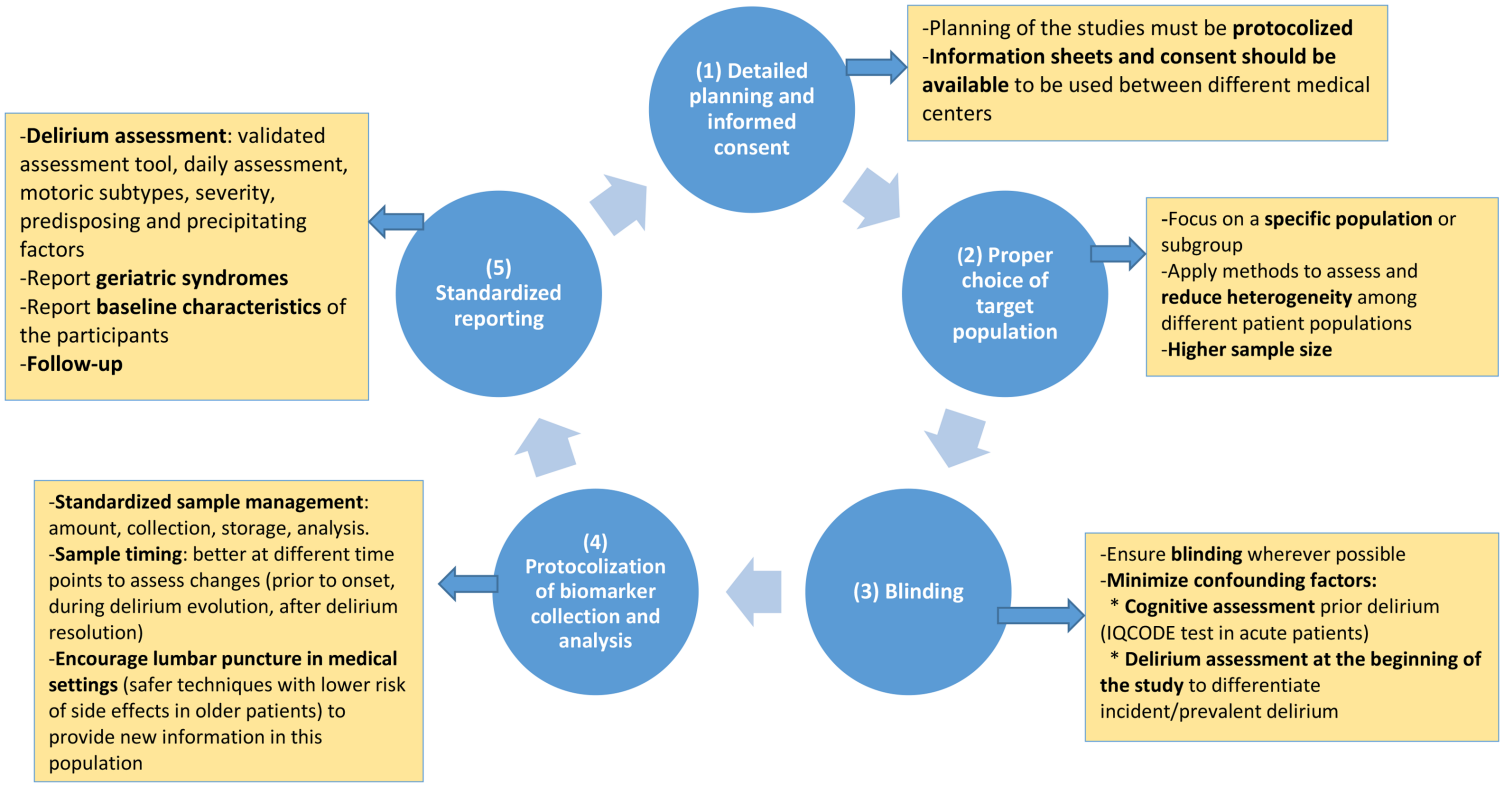
Recommendations for future research in delirium biomarkers.

Based on the limitations of the studies included in this systematic review and meta-analysis, we identified five major action areas to advance this research, designing better quality studies: (1) detailed planning and informed consent; (2) proper choice of target population; (3) blinding; (4) protocolization of biomarker collection and analysis; and (5) standardized reporting, which are summarized in Figure 3. and could be useful to improve the “Core Outcome Set” (COMET) in the field of delirium (Williamson et al., 2017).

## Conclusion

The relevance of delirium in older patients, both for its long- and short-term adverse consequences, provides a compelling reason to investigate its pathophysiology to prevent its appearance and improve the approach.

Despite the recent surge in studies on delirium biomarkers, there is not consistent evidence in the obtained results given the great heterogeneity that reflects the complexity of its pathophysiological mechanisms. Our data identified a significant association between biomarkers of neuroinflammation, such as CRP, IL-6, or TNF-*α*, and the risk of delirium, but further research is needed to standardize the methodology, improve scientific evidence and translate the current knowledge into pragmatic tools for routine clinical practice in the field of delirium.

## Author contributions

LL-V, BC-V, and NM-V: conception and design of the work. LL-V and BC-V: acquisition of data. AG-H: analysis. AG-H and LL-V: interpretation of data. LL-V, NM-V, and RR-O: drafting of manuscript. JF-I, ES, FZ-F, MS, ÁM-V, and MI: critical revision. All authors contributed to the article and approved the submitted version.

## Conflict of interest

The authors declare that the research was conducted in the absence of any commercial or financial relationships that could be construed as a potential conflict of interest.

## Publisher’s note

All claims expressed in this article are solely those of the authors and do not necessarily represent those of their affiliated organizations, or those of the publisher, the editors and the reviewers. Any product that may be evaluated in this article, or claim that may be made by its manufacturer, is not guaranteed or endorsed by the publisher.
