# Supplementary material for: Biomarkers of delirium risk in older adults: a systematic review and meta-analysis
Source: Front Aging Neurosci. 2023 May 12;15:1174644. doi: 10.3389/fnagi.2023.1174644 (PMC10213257; doi:10.3389/fnagi.2023.1174644)
Supplement: Supplementary file 1 [file Data_Sheet_1.DOCX]

Supplementary Material


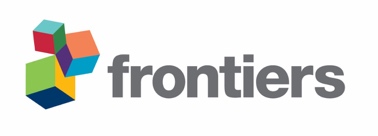
Biomarkers of delirium risk in older adults:

a systematic review and meta-analysis

Lozano-Vicario, Lucía^1*^; García-Hermoso, Antonio^2^; Cedeño-Veloz, Bernardo Abel^1^; Fernández-Irigoyen, Joaquín^3^; Santamaría, Enrique^3^; Romero-Ortuno, Román^4^; Zambom-Ferraresi, Fabricio^2,5^; L. Sáez de Asteasu, Mikel^2^; Muñoz-Vázquez, Ángel Javier^6^; Izquierdo, Mikel^2^; Martínez-Velilla, Nicolás^1,2,5^

*** Correspondence:**

Corresponding Author: Lozano-Vicario, Lucía lucia.lozanovicario@gmail.com

***Supplementary material 1. Search Strategies***

**MEDLINE:**

1. “delirium”[MeSH Terms] OR “delirium”[All Fields] OR “acute confusional state”[All fields] OR “acute brain failure”[All fields] OR “acute confusion”[All Fields] OR “postoperative delirium”[All Fields] OR “POD”[All Fields] OR “postoperative cognitive disorder”[All fields] OR “postoperative cognitive dysfunction”[All Fields] OR “POCD”[All Fields] OR “postoperative cognitive impairment”[All Fields]
2. “biomarkers” [MeSH Terms] OR “biomarkers” [All Fields] OR “biomarker” [All Fields]
3. 1 AND 2

(“delirium”[MeSH Terms] OR “delirium”[All Fields] OR “acute confusional state”[All fields] OR “acute brain failure”[All fields] OR “acute confusion”[All Fields] OR “postoperative delirium”[All Fields] OR “POD”[All Fields] OR “postoperative cognitive disorder”[All fields] OR “postoperative cognitive dysfunction”[All Fields] OR “POCD”[All Fields] OR “postoperative cognitive impairment”[All Fields]) AND (“biomarkers” [MeSH Terms] OR “biomarkers” [All Fields] OR “biomarker” [All Fields]) 🡪 **1202 hits**

**EMBASE:**

1. “delirium”/exp OR delirium OR “postoperative delirium” OR “postoperative cognitive dysfunction” OR “postoperative cognitive decline” (41955 results)
2. Biomarker OR biomarkers OR “biological marker” (618714)
3. 1 AND 2 🡪 **701 hits**

**The Cochrane Library:**

1. Delirium OR postoperative delirium OR postoperative cognitive dysfunction OR postoperative cognitive disorder
2. Biomarker
3. 1 + 2 🡪 **43 hits**

**Scopus:**

1. Delirium
2. Biomarker
3. 1+2 🡪 **415 hits**

**Web of Science:**

1. Delirium
2. Biomarker
3. 1+2🡪 **171 hits**
