# Supplementary material for: Biomarkers of delirium risk in older adults: a systematic review and meta-analysis
Source: Front Aging Neurosci. 2023 May 12;15:1174644. doi: 10.3389/fnagi.2023.1174644 (PMC10213257; doi:10.3389/fnagi.2023.1174644)
Supplement: Supplementary file 2 [file Data_Sheet_2.DOCX]

Supplementary Material


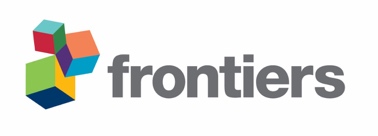
Biomarkers of delirium risk in older adults:

a systematic review and meta-analysis

Lozano-Vicario, Lucía^1*^; García-Hermoso, Antonio^2^; Cedeño-Veloz, Bernardo Abel^1^; Fernández-Irigoyen, Joaquín^3^; Santamaría, Enrique^3^; Romero-Ortuno, Román^4^; Zambom-Ferraresi, Fabricio^2,5^; L. Sáez de Asteasu, Mikel^2^; Muñoz-Vázquez, Ángel Javier^6^; Izquierdo, Mikel^2^; Martínez-Velilla, Nicolás^1,2,5^

*** Correspondence:**

Corresponding Author: Lozano-Vicario, Lucía lucia.lozanovicario@gmail.com

***Supplementary data 2. Summary of assessment of risk of bias and additional comments on study quality***

| **Study (year)**  **Biomarker(s)** | **Selection of participants** | **Confounding variables (dementia)** | **Measurement of exposure (delirium)** | **Blinding of outcome assessments** | **Incomplete outcome data** | **Selective outcome reporting** | **Comments** |
| --- | --- | --- | --- | --- | --- | --- | --- |
| Adam et al.  (2020)  AChE  BChE | Low  Consecutive | High  Dementia was not assessed | Low  CAM-ICU | High | Low | Low | Delirium was excluded at the beginning of the study, but dementia was not measured |
| Adamis et al.  (2007)  APOE 4, IL-1 (IL-1a, IL-1b, IL-1ra), IL-6, TNF- α, IGF-I, IFN- γ, LIF, CRP | Low  Consecutive | Low  MMSE | Low  CAM | High | Low | Low | Delirium was not excluded at the beginning of the study |
| Adamis et al.  (2009)  APOE 4, IL-1 (IL-1α, IL-1β, IL-1RA), IL-6, TNF-α, IFN-γ, LIF, IGF-I | Low  Consecutive | Low  MMSE | Low  CAM | High | Low | Low | Delirium and dementia were not excluded at the beginning of the study |
| Altmimi et al. (2016)  S100 | Low  Consecutive | Low  MMSE | Low  CAM and CAM-ICU | High | Low | Low | Delirium and dementia were excluded at the beginning of the study |
| Avila-Funes et al. (2015)  Estradiol and cortisol | Low  Consecutive | Low  MMSE | Low  CAM and DSM-IV | High | Low | Low | Delirium and dementia were not excluded at the beginning of the study |
| Bakker et al.  (2012)  Creatinine | Low  Consecutive | Low  MMSE | Low  CAM-ICU | High | Low | Low | Preoperative delirium was excluded |
| Ballweg et al.  (2021)  Tau, IL-1RA, IL-2, IL-4, IL-6, IL-8, IL-10, IL-12, GFAP, MCP-1, TNF-α, NfL | Low  Consecutive | High  Only documented dementia | Low  CAM, CAM-ICU | High | Low | High | Only documented dementia was excluded  Delirium was not excluded |
| Baranyi et al.  (2012)  Austria | Low  Consecutive | Low  Short cognitive Performance test  Documented dementia was excluded | Low  DRS | Low  A nurse and a psychiatrist evaluated delirium daily | Low | High | DRS was the only tool used in this study  CRP was also analyzed but results were not reported |
| Baranyi et al.  (2013)  NSE, S100B | Low  Consecutive | Low  Short cognitive Performance test  Documented dementia was excluded | Low  DRS | Low  A nurse and a psychiatrist evaluated delirium daily | High | High | NSE and S100B were correlated with cognitive impairment but information about delirium was not reported |
| Baranyi et al.  (2014)  sIL-2R | Low  Consecutive | Low  Short cognitive Performance test  Documented dementia was excluded | Low  DRS | Low  A nurse and a psychiatrist evaluated delirium daily | Low | High | DRS was the only tool used in this study |
| Beaubien-Souligny et al. (2020)  NETs, IL-6, CRP, myeloperoxidase, citrullinated histone 3, and pentraxin-related protein 3 | Low  Consecutive | High  No diagnostic | High  No delirium screening tool | High | High | High | The sex of the patients was not reported  No information about delirium was reported  There was not follow up |
| Beloosesky et al. (2004)  CRP, fibrinogen, ESR | Low  Consecutive | Low  MMSE | Low  CAM | High | High | High | Delirium was not excluded at the beginning of the study |
| Bisschop et al. (2011)  Cortisol, insulin, glucose | Low  Consecutive | Low  IQCODE | Low  CAM | Low  The presence or absence of delirium was assessed by a physician and a nurse (separately) | Low | Low | Delirium was not excluded at the beginning of the study |
| Brum et al. (2015)  BDNF, TNF-α | High  For each oncology inpatient with delirium included in the study, the team searched for an oncology inpatient without delirium and a healthy control matched according to age, education, and sex | Low  MMSE | Low  CAM | High | High | Low | Delirium was not excluded at the beginning of the study |
| Cape et al. (2014)  IL-1β, IL-1ra, IFN-γ, IGF-1, GFAP | Low  Consecutive | Low  IQCODE | Low  CAM | High | Low | Low | In N=24, blood samples were taken at the same time as CSF  Delirium was not excluded at the beginning of the study |
| Caplan et al. (2017)  Apolipoprotein E | Low  Consecutive | Low  MMSE, IQCODE | Low  CAM, Delirium Index | High | Low | Low | Geriatric inpatients with delirium (cases) were compared to geriatric outpatients with AD without delirium (controls) |
| Casey et al. (2019)  IL-1β, IL-1ra, IL-2, IL-4, IL-6, IL-8, IL-10, IL-12p70, MCP-I, TNF-α, NfL | Low  Consecutive | High  Only documented dementia was excluded | Low  3D-CAM; CAM-ICU | High | Low | Low | Delirium was not excluded at the beginning of the study |
| Cerejeira et al. (2011)  AChE, BChE | Low  Consecutive | Low  MMSE | Low  CAM | Low  Positive cases of delirium (according to CAM criteria) were confirmed with DSM-IV-TR criteria. | Low | Low | Delirium was excluded before the beginning of the study |
| Cerejeira et al. (2011)  CRP, IL-1β, TNF-α, IL-6, IL-8, IL-10 | Low  Consecutive | Low  MMSE | Low  CAM | Low  Positive cases of delirium (according to CAM criteria) were confirmed with DSM-IV-TR criteria. | Low | Low | Delirium was excluded before the beginning of the study |
| Chan et al.  (2021)  Aβ40, Aβ42, t-tau, p-tau | Low  Consecutive | Low  MMSE | Low  CAM, DRS | Low  Study team members involved in assessing delirium were blinded to GDS-15 scores. | High | Low | Delirium was excluded before the beginning of the study |
| Chen et al. (2020)  miR-210, CRP, IL-6, TNF-α | Low  Consecutive | High  No diagnostic | Low  DSM-V | Low  An experienced psychiatrist who was blind to this study was invited to perform the POD assessments among the enrolled patients | Low | High | No information about IL-6, TNF-α |
| Chen et al. (2019)  IL-6 | Low  Consecutive | Low  MMSE | Low  CAM-ICU | High | Low | Low | Delirium was excluded before the beginning of the study |
| Chu et al.  (2016)  IGF-I | Low  Consecutive | Low  MMSE | Low  CAM | Low  Delirium was diagnosed by a research psychiatrist according to the DSM-IV criteria | Low | Low | Delirium was excluded before the beginning of the study |
| Cinar et al. (2014)  CRP, IGF-I, TNF-α | Low  Consecutive | Low  MMSE | Low  DRS | High | High | High | Delirium was excluded before the beginning of the study |
| Conti et al.  (2020)  DBI, IL-17, IL-6, TNF-α | Low  Consecutive | Low  MMSE | Low  DSM-IV criteria and 4AT | High | High | Low | Geriatric inpatients with delirium (cases) were compared to geriatric outpatients with AD without delirium and with healthy controls. |
| Cooper et al.  (2020)  NfL, t-tau, ubiquitin carboxy- terminal hydrolase L1 and GFAP | Low  Consecutive | High, no diagnostic | Low  ICDSC | High | High | Low | Covid-19 patients were compared with ICU non-covid-19 patients.  Delirium was not excluded before the beginning of the study |
| Cunningham et al. (2018)  APOE 4, Aβ42, t-tau and p-tau | Low  Consecutive | Low  MMSE | Low  CAM | High | Low | Low | Delirium was not excluded before the beginning of the study |
| Dejonghe et al. (2012)  Tryptophan, kynurenine and IDO activity | Low  Consecutive | Low  IQCODE | Low  CAM | Low  Delirium was scored during weekdays separately by a physician and a nurse. When the diagnosis of delirium was doubtful, the patient was discussed with the geriatric consultation team to gain a consensus | Low | Low | Delirium was not excluded before the beginning of the study |
| Dillon et al.  (2016)  CRP | Low  Consecutive | Low  MMSE | Low  CAM | Low  The presence of delirium by chart review was adjudicated by a minimum of two delirium experts, and discordance was resolved through consensus | Low | Low | Delirium was excluded at the beginning of the study |
| Egberts et al.  (2015)  Neopterin, IL-6, IGF-I | Low  Consecutive | Low  MMSE | Low  DRS | Low  When the diagnosis of delirium was doubtful, the case was discussed with the geriatric consultation team to gain consensus | Low | Low | Delirium was not excluded at the beginning of the study |
| Egberts et al.  (2017)  CRP, NLR | High  Opportunistic | High | Low  DSM-IV criteria | High | Low | Low | Retrospective study  Delirium was not excluded at the beginning of the study |
| Egberts et al. (2019)  Neopterin, CRP, aminoacids, HVA, eGRF | Low  Consecutive | Low  MMSE | Low  DSM-IV criteria and CAM | High | Low | Low | This is a subanalysis of two different cohorts (medical and surgical patients). Preoperative delirium was excluded in DECO study |
| Ehler, Petzold et al. (2019)  Nf | Low  Consecutive | High  Only documented dementia was excluded | Low  CAM-ICU | High | High | High | Little data is available about delirium, this study focuses on SAE |
| Ehler, Saller et al. (2019)  NT-proCNP, NSE, S100B | Low  Consecutive | High  Only documented dementia was excluded | Low  CAM- ICU | High | High | High | Little data is available about delirium, this study focuses on SAE |
| Elsamadicy et al. (2018)  Hb | High  Opportunistic | High  No diagnostic | High  No tool validated | High | High | High | Retrospective study  Delirium was not excluded at the beginning of the study |
| Fernandez et al. (2018)  RDW | High  Opportunistic | High  No diagnostic | High  No tool validated | High | High | High | Retrospective study  No data available about delirium |
| Fong et al. (2020)  t-tau, GFAP, NfL, UCHL-1 | Low  Consecutive | Low  MMSE and other tests | Low  CAM | Low | Low | Low | Subanalysis of SAGES study (case-control)  Delirium was excluded at the beginning of the study |
| Fong et al.  (2021)  p-tau, t-tau, Aβ40, Aβ42 | Low  Consecutive | Low  MMSE and GCP | Low  CAM | Low | Low | Low | Delirium was not excluded at the beginning of the study |
| Ford et al.  (2013)  Vitamin D | High  Opportunistic | High  Dementia was not excluded | Low  ICD-10 | High | High | High | Retrospective study  Delirium and dementia were not excluded at the beginning of this study |
| Forget et al.  (2021)  CRP | High  Opportunistic | High  Dementia was not excluded | Low  DSM-V criteria | High | High | High | Retrospective study  Delirium and dementia were not excluded at the beginning of this study |
| Gailiusas et al. (2019)  NSE, GFAP | Low  Consecutive | High  Documented dementia was excluded but no informant history, MMSE or IQCODE | Low  CAM-ICU | High | Low | Low | Delirium was not excluded at the beginning of this study |
| Girard et al.  (2012)  CRP, MMP-9, MPO, NGAL, sTNFR1, D-dimer, protein C, PAI-1, VWF | Low  Consecutive | High  Dementia was not excluded | Low  CAM-ICU | High | Low | Low | Delirium and dementia were not excluded at the beginning of this study |
| Halaas et al.  (2019)  NfL | Low  Consecutive | Low  IQCODE | Low  CAM | High | Low | Low | 2 cohorts (314 patients with hip fracture and 172 healthy controls with elective surgery). Delirium was not excluded at the beginning of the study |
| Halaas et al.  (2021)  Neurogranin | Low  Consecutive | Low  IQCODE | Low  CAM | High | Low | Low | 3 cohorts (128 patients with hip fracture, 127 with elective surgery and 46 AD patients)  Delirium and dementia were excluded at the beginning of the study |
| Hall et al.  (2013)  S100B | Low  Consecutive | Low  MMSE and IQCODE | Low  CAM | High | Low | Low | Delirium was not excluded at the beginning of the study |
| Han, Zhang et al. (2020)  Metabolomics and lipidomics | Low  Consecutive | Low  MMSE | Low  CAM-Chinese revision | High | Low | Low | Delirium and dementia were excluded at the beginning of the study  Total N=80 (delirium in 15 cases) but 10 delirium were selected and compared with 30 non-delirium patients |
| Han, Chen et al. (2020)  Proteomics | Low  Consecutive | Low  MMSE | Low  CAM-Chinese revision | High | Low | Low | Delirium and dementia were excluded at the beginning of the study  Total N=80 (delirium in 15 cases) but 10 delirium were selected and compared with 30 non-delirium patients |
| Henjum et al. (2018)  sTREM2, t-tau, p-tau, Aβ42 | Low  Consecutive | Low  ICD-10 criteria | Low  CAM | High | Low | Low | Delirium and dementia were not excluded at the beginning of the study.  A hip fracture cohort was compared to a medical delirium cohort. |
| Hirsch et al. (2016)  IFN-γ, IL-10, IL-12p70, IL-2, IL-4, IL-5, IL-6, IL-8, TNF-α, MCP-1, MIP-1α, MIP-1β, Aβ-40, Aβ-42, RAGE, Calprotectin MRP8/14 | Low  Consecutive | High  Documented dementia was excluded but no informant history, MMSE or IQCODE | Low  CAM | High | Low | Low | Biomarkers of POD (only one patient in the study developed delirium) were analyzed with biomarkers of POCD. |
| Hov 2017 et al. (2017)  S100B and p-tau | Low  Consecutive | Low  IQCODE, MMSE | Low  CAM | Low  Information from nurses, close relatives and hospital records in combination with 10 to 30 min interview with the participant | Low | Low | 2 cohorts:  -Hip fracture patients (delirium was not exclude preoperatively but prevalent/incident cases were reported)  -Elective major surgery (delirium and dementia were excluded at the beginning of the study) |
| Ida et al. (2020)  NLR, PWR, PLR | High  Retrospective | High  Only documented dementia was excluded | High  Chart-based method | High | Low | Low | Retrospective study.  Data were collected using medical records |
| Idland et al. (2017)  Aß42, t-tau, p-tau | Low  Consecutive | Low  IQCODE | Low  CAM | High | Low | Low | Delirium and dementia were not excluded at the beginning of the study |
| Inoue et al. (2017)  pNF-H | Low  Consecutive | High  Only patients with slight or more clinically relevant cognitive dysfunction before surgery were excluded | Low  CAM-ICU | Low  Nurses assessed delirium-associated symptoms at least three times a day.  Suspected POD was confirmed by the investigators according to CAM-ICU | Low | High | Delirium was not excluded at the beginning of the study. Characteristics of the control group are not reported. |
| Kalantar et al. (2018)  mRNA | Low  Consecutive | Low  IQCODE | Low  CAM | High | Low | Low | Delirium and dementia were not excluded at the beginning of the study |
| Kalyoncuoglu et al. (2020)  CAR | Low  Consecutive | High  Only significant mental impairment was excluded | Unclear risk  CAM-ICU and then if the RASS was -3 or greater | High | Low | Low | Delirium was not excluded at the beginning of the study |
| Katsumi et al. (2020)  CRP, IL-6 and CHI3L1 | Low  Consecutive | Low  General cognitive performance tests | Low  CAM and CAM-S | High | Low | Low | Dementia and delirium were excluded at the beginning of the study |
| Kazmierski et al. (2021)  hsCRP and MCP-1 | Low  Consecutive | Low  MMSE | Low  CAM-ICU | High | Low | Low | Dementia and delirium were excluded at the beginning of the study |
| Kazmierski et al. (2013)  IL-2 and TNFα | Low  Consecutive | Low  MoCA  TMT-B | Low  CAM-ICU | High | Low | Low | Dementia and delirium were not excluded at the beginning of the study |
| Knaak et al. (2019)  CRP | Low  Consecutive | Low  MMSE | Low  DSM-IV criteria | High | Low | Low | Delirium was not excluded at the beginning of the study but patients with MMSE > or = 24 were excluded |
| Kotfis, Olejnik et al. (2019)  NLR | Low  Consecutive | High  No diagnostic | Low  CAM-ICU | High | Low | Low | Dementia and delirium were not excluded at the beginning of the study |
| Kotfis, Slozowska et al. (2019)  PLR and PWR | Low  Consecutive | Low  MMSE | Low  CAM-ICU | Low  The team of nurses, anesthesiologists, intensivists and cardiac surgeons were involved in this process. The final delirium diagnosis was made by consultant neurologist using the DSM-V criteria | Low | Low | Dementia and delirium were excluded at the beginning of the study |
| Kozak et al. (2016)  IL-1β, IL-18, TNF-α, BDNF, NSE | Low  Consecutive | High  No diagnostic | Low  DSM-V criteria | High | Low | Low | Dementia and delirium were not excluded at the beginning of the study |
| Li et al.  (2017)  Leptin | Low  Consecutive | High  Only documented history of severe dementia was excluded | Low  CAM-ICU | High | Low | Low | Delirium was excluded at the beginning of the study |
| Lin et al. (2020)  ChAT, AChE, BuChE, IL-6, TNF-α | Low  Consecutive | Low  MMSE | Low  CAM | High | Low | Low | Delirium was not excluded at the beginning of the study but patients with MMSE<23 were excluded |
| Lu et al. (2020)  TP | Low  Consecutive | High  Only documented history of dementia was excluded | Low  CAM | High | Low | Low | Delirium and dementia were not excluded at the beginning of the study |
| Ma et al. (2020)  Ach and cortisol | Low  Consecutive | High  Only previous history of mental illness was excluded | Low  DSM criteria | Low  2 independent psychiatrists. If the result was inconsistent, delirium was evaluated by a 3^rd^ psychiatrist | Low | Low | Delirium was excluded at the beginning of the study |
| McNeil et al. (2019)  IL-6, IL-8, sTNFR1, Protein C, PAI-1 and S100B | Low  Consecutive | Low  IQCODE | Low  Brief confusion assessment method | High | Low | Low | Delirium was not excluded at the beginning of the study. The primary outcome of this study was delirium duration |
| Menzenbach et al. (2021)  IL-3, IL-8, IL-10, Cripto, CCL2, RAGE, CXCL5, uPAR, ANGPT2, TIE2, THBD, SDC1, E-Selectin, VCAM-1, ICAM-1, NSE | Low  Consecutive | High  No diagnostic | Low  CAM, CAM-ICU, 4AT and DOS | High | Low | Low | Delirium and dementia were not excluded at the beginning of the study |
| Miao et al. (2016)  Neopterin, CRP, IL-6, IGF-1 | Low  Consecutive | Low  MMSE | Low  DSM-IV criteria | High | Low | Low | Delirium was not excluded at the beginning of the study |
| Michels et al. (2021)  AchE and BChE | Low  Consecutive | High  No diagnostic | Low  NU-DESC | High | High | High | Delirium and dementia were not excluded at the beginning of the study  No specific results about delirium are reported (delirium is included in the “complication group” with infections, heart failure, renal failure…) |
| Mietani et al. (2019)  pNF-H, ICAM-1, PECAM-1, VCAM, E-selectin, P-selectin, IL-6, IL-1ß, TNF-α | Low  Consecutive | High  Only clinically relevant cognitive impairment was excluded | Low  CAM-ICU | High | Low | Low | Delirium and dementia were not excluded at the beginning of the study (no diagnosis of cognitive status before surgery was reported) |
| Mossanen et al. (2020)  WBC (CD 3, CD 4, CD8, CD 14, CD 16, CD 19, CD 45, CD 56), IL-1ß, IL-2, IL-4, IL-6, IL-8, IL-10, IL-12, IL-17 | Low  Consecutive | High  No diagnostic | Low  CAM-ICU | High | High | High | Delirium and dementia were not excluded at the beginning of the study  No specific results about delirium are reported (delirium is included in the “complication group” with infections, heart failure, renal failure…) |
| Neerland et al. (2016)  CRP, IL-6, sIL-6R | Low  Consecutive | Low  IQCODE | Low  CAM, MDAS | High | Low | Low | Delirium and dementia were not excluded at the beginning of the study, but they were registered and subanalysis were done (patients with/without cognitive impairment; patients with incident/prevalent delirium) |
| Neerland et al. (2020)  FABP3 | Low  Consecutive | Low  IQCODE, MMSE | Low  CAM | High | Low | Low | Delirium was excluded in the healthy control group (in hip fracture patients delirium was measured preoperatively and assessed as incident or prevalent).  Dementia was only excluded in the healthy control group (IQCODE was used in hip fracture patients to assess cognitive impairment at the beginning of the study) |
| Nguyen et al. (2014)  Cortisol and S100B | Low  Consecutive | High  Only documented dementia was excluded | Low  CAM-ICU, RASS | High | Low | Low | Brain dysfunction in measured with CAM-ICU, Glasgow coma scale and RASS scale  Delirium was not excluded at the beginning of the study |
| Nguyen et al. (2016)  Prolactin | Low  Consecutive | High  Only documented dementia was excluded | Low  CAM-ICU, RASS | Low | Low | Low | Delirium was not excluded at the beginning of the study  The person that measured delirium was not aware of prolactin results |
| Osse et al. (2012)  Neopterin and HVA | Low  Consecutive | Low  MMSE | Low  CAM-ICU | High | Low | Low | Delirium and dementia were excluded at the beginning of the study |
| Pan et al. (2019)  Spermidine, glutamine, putrescine, Aß-42, t-tau and p-tau | Low  Consecutive | Low  MMSE | Low  CAM | High | Low | Low | Delirium was not excluded at the beginning of the study |
| Parker et al. (2021)  Aß40, Aß42, p-tau, t-tau, NfL | Low  Consecutive | High  Only documented dementia was excluded | Low  CAM or 3D-CAM | High | Low | Low | Delirium was evaluated preoperatively (all the patients were non-delirious before surgery) |
| Peng et al. (2019)  TNF-α, CRP, CAR, IL-6 | Low  Consecutive | Low  MMSE | Low  DSM V criteria | Low  An experienced psychiatrist who was blinded to this study was consulted to confirm POD | Low | Low | Delirium was not excluded at the beginning of the study but MMSE<24 was a exclusion criteria |
| Pfister et al. (2008)  CRP, IL-6, S100B, cortisol | Low  Consecutive | High  Only documented dementia as excluded | Low  CAM-ICU | High | Low | Low | Delirium attributable to a cause other than sepsis was excluded |
| Pilling et al. (2020)  Vitamin D | Low  Consecutive | High  No cognitive assessment was done at the beginning of the study | Low  ICD-10 criteria | High | Low | Low | Patients with previous delirium was excluded at the beginning of the study |
| Plaschke et al. (2010)  Cortisol and IL-6 | Low  Consecutive | High  Only documented dementia was excluded | Low  CAM-ICU | High | Low | Low | Delirium was not excluded at the beginning of the study |
| Plaschke et al. (2016)  AChE and BuChE | Low  Consecutive | High  Only documented neurological problems were excluded | Low  Nu-DESC | High | Low | Low | Delirium was not excluded at the beginning of the study |
| Pol et al. (2014)  CRP | Low  Consecutive | High  Only GFI is done | Low  DSM-IV | Low  Nurse and geriatrician | Low | Low | Delirium and dementia were not excluded at the beginning of the study |
| Poljak et al. (2014)  Proteomics | Low  Consecutive | Low  IQCODE, MMSE | Low  CAM | High | Low | Low | Delirium was not excluded but measured before surgery  Dementia and delirium were excluded in control group |
| Rasmussen et al. (2000)  NSE and S100B | Low  Consecutive | High  Not reported | Low  DSM III criteria | High | High | Low | Delirium and dementia were not excluded at the beginning of the study |
| Reznik et al. (2021)  NLR, glucose and troponin | Low  Consecutive | High  Not reported | Low  DSM criteria | High | Low | Low | Retrospective study  Delirium and dementia were not excluded at the beginning of the study |
| Ritchie et al. (2014)  CRP | Low  Consecutive | High  Not reported | Low  CAM | High | Low | Low | Delirium and dementia were not excluded at the beginning of the study |
| Rudolph et al. (2008)  Chemokines and cytokines | Low  Consecutive | Low  MMSE | Low  CAM | High | Low | Low | Preoperative delirium was excluded at the beginning of the study. Mental status was assessed at the beginning of the study, to evaluate cognitive impairment |
| Saito et al. (2020)  DNAm | Low  Consecutive | Low  MoCA | Low  CAM-ICU | High | Low | Low | Delirium and dementia were not excluded at the beginning of the study |
| Sajjad et al. (2020)  Norway | Low  Consecutive | Low  ICD-10 criteria of dementia, MMSE | Low  CAM | High | Low | Low | Delirium and dementia were not excluded at the beginning of the study in hip fracture group.  Delirium and dementia were excluded in elective surgery group.  Patients with AD dementia were included in dementia group. |
| Saller et al. (2019)  NfL, tau and GFAP | Low  Consecutive | Low  MMSE | Low  CAM-ICU | Low  CAM-ICU 3 times daily by nurses and twice per day by the study team (and completed with a chart review) | Low | Low | Dementia was excluded at the beginning of the study. |
| Saller et al. (2020)  ANP, NT-proCNP | Low  Consecutive | High | High  Delirium was assessed retrospectively | Low (5 specialists) | Low | Low | Delirium and dementia were not excluded at the beginning of the study |
| Sanchez et al. (2013)  Leptin | Low  Consecutive | Low  MMSE | Low  CAM and DSM IV criteria | High | Low | Low | Dementia was excluded at the beginning of the study, but delirium was not. |
| Sasajima et al. (2000)  Albumin | Low  Consecutive | Low  HDS-R | Low  CAM | High | Low | Low | Delirium and dementia were not excluded at the beginning of the study |
| Shen et al. (2016)  IGF-I, CRP and IL-6 | Low  Consecutive | Low  MMSE | Low  CAM and DRS-R-98 | Low (psychiatrist DSM criteria) | Low | Low | Delirium and dementia were excluded at the beginning of the study |
| Shen et al. (2020)  Melatonin | Low  Consecutive | Low  MMSE | Low  CAM | High | Low | Low | Delirium was not excluded at the beginning of the study |
| Simons et al. (2018)  Tau, amyloid, adiponectin, neopterin, IL-1ß, IL-6, IL-10, TNF-α | Low  Consecutive | High  Only documented dementia was excluded | Low  CAM-ICU | High | Low | Low | Delirium was excluded at the beginning of the study |
| Sugita et al. (2018)  Glucose, HbA1C, CRP, creatinine, NT-proBNP, LDL-C, TC, TG, HDL-C | Low  Consecutive | High  Dementia was not excluded | High  Intensive Care Delirium Screening Checklist | High | Low | Low | Delirium was excluded at the beginning of the study |
| Sun et al. (2016)  Cortisol, IL-6, procalcitonin, CRP, AßI-40 | Low  Consecutive | Low  MMSE | Low  CAM | High | Low | Low | Dementia was excluded at the beginning of the study, but delirium was not |
| Szwed et al. (2020)  GFAP, NSP, pNFH, VILIP-1 | Low  Consecutive | Low  MMSE | Low  CAM-ICU | High | Low | Low | Delirium was not excluded at the beginning of the study |
| Szwed et al. (2021)  microRNA-1-3p, microRNA-21-5p, GFAP, NSP, pNFH, VILIP-1 | Low  Consecutive | Low  MMSE | Low  CAM-ICU | High | Low | Low | Delirium was not excluded at the beginning of the study |
| Thisayakorn et al. (2021)  WBC, NLR, MPV, p02, bicarbonate | Low  Consecutive | High  Only documented dementia was excluded | Low  CAM-ICU | High | Low | Low | Delirium was not excluded at the beginning of the study |
| Thomas et al. (2008)  SAA | Low  Consecutive | Low  MMSE, IQCODE | Low  CAM (but only in one moment) | High | Low | Low | Delirium and dementia were measured but not excluded |
| Van Munster, Korevaar et al. (2009)  S100B | Low  Consecutive | Low  MMSE, IQCODE | Low  CAM | Low  Separately by a physician and a nurse | Low | Low | Delirium and dementia were assessed but not excluded |
| Van den Boogaard, Kox et al. (2011)  TNFα, IL1ß, IL-6, IL-8, IL-17, IL-18, MIF, IL-1RA, IL-10, MCP-1, CRP, PCT, S100ß, Aß1, Aß40, Aß42, t-tau, S100ß, cortisol | Low  Consecutive | High  Only documented dementia was excluded | Low  CAM-ICU | High | Low | Low | Delirium was not excluded at the beginning of the study |
| Van den Boogaard, van Swelm et al. (2011)  Urine proteomics | Low  Consecutive | High  Dementia was not excluded | Low  CAM-ICU | High | Low | Low | Delirium and dementia were not excluded at the beginning of the study |
| Van Munster, Korse et al. (2009)  S100B, NSE, IL-6, IL-8 | Low  Consecutive | Low  IQCODE-SF | Low  CAM | Low  Separately by a physician and a nurse | Low | Low | Delirium and dementia were assessed but not excluded |
| Van Munster et al. (2010)  Cortisol, IL-6, IL-8, S100B | Low  Consecutive | Low  IQCODE-SF | Low  CAM | Low  Separately by a physician and a nurse | Low | Low | Delirium and dementia were assessed but not excluded |
| Van Munster et al. (2011)  GFAP, IL-1ß, IL-6, ß-amyloid, tau | High  Retrospective | Low  IQCODE-SF | Low  CAM | Low  DSM IV criteria and DOS | Low | Low | Dementia was not excluded. |
| Van Munster et al. (2012)  SAA, IL-6, cortisol | Low  Consecutive | Low  IQCODE-SF | Low  CAM | Low  Separately by a physician and a nurse | Low | Low | Delirium and dementia were assessed but not excluded |
| Vasunilashorn et al. (2018)  Proteomics | Low  Consecutive | Low  Neurocognitive battery | Low  CAM | High | Low | Low | Delirium and dementia were excluded at the beginning of the study |
| Vasunilashorn et al. (2021)  Proteomics | Low  Consecutive | Low  MMSE | Low  CAM | High | Low | Low | Delirium and dementia were excluded at the beginning of the study |
| Wu, Xu et al. (2017)  TRX | Low  Consecutive | Low  MMSE | Low  CAM | High | Low | Low | Delirium and dementia were excluded at the beginning of the study |
| Wyrobek et al. (2017)  BDNF | Low  Consecutive | Low  MMSE | Low  CAM and CAM-ICU | High | Low | Low | Delirium and dementia were excluded at the beginning of the study |
| Yuan et al. (2020)  Exosome α-synuclein release, IL1ß, IL-6, TNFα | Low  Consecutive | Low  MMSE | Low  CAM | High | Low | Low | Delirium and dementia were excluded at the beginning of the study |
| Zhao et al. (2018)  SAA | Low  Consecutive | Low  MMSE | Low  CAM-ICU | High | Low | Low | Delirium was not excluded at the beginning of the study, but dementia was |

**Newcastle-Ottawa** **quality assessment scale**

1. **For evaluation of cohort studies**

| **Author** | **Selection** | | | | **Comparability** | | **Outcome** | | | **Total** |
| --- | --- | --- | --- | --- | --- | --- | --- | --- | --- | --- |
| First author, year | #1 | #2 | #3 | #4 | #5 | #6 | #7 | #8 | #9 |  |
| Adam et al., 2020 | * | * | * | * | * | * |  | * |  | 7 |
| Adamis et al., 2007 | * | * | * |  |  |  |  | * |  | 4 |
| Adamis et al., 2009 | * | * | * |  |  |  |  | * |  | 4 |
| Altmimi et al., 2016 | * | * | * | * | * | * |  | * |  | 7 |
| Avila-Funes et al., 2015 | * | * | * |  | * | * |  | * | * | 7 |
| Bakker et al., 2012 | * | * | * | * | * | * | * | * |  | 8 |
| Ballweg et al., 2021 | * | * | * |  | * | * | * | * | * | 8 |
| Baranyi et al., 2012 | * | * | * | * | * | * | * | * |  | 8 |
| Baranyi et al., 2013 | * |  | * | * |  |  | * | * |  | 5 |
| Baranyi et al., 2014 | * | * | * | * | * | * | * | * |  | 8 |
| Beaubien-Souligny et al., 2020 |  |  |  |  | * |  |  |  |  | 1 |
| Beloosesky et al., 2004 | * |  | * |  |  |  | * | * |  | 4 |
| Bisschop et al., 2011 | * | * | * |  | * | * | * | * | * | 8 |
| Brum et al., 2015 | * | * | * |  | * | * | * |  |  | 6 |
| Cape et al., 2014 | * | * | * | * | * | * | * |  |  | 7 |
| Caplan et al., 2017 | * | * | * | * | * | * |  | * | * | 8 |
| Casey et al., 2019 | * | * | * |  | * |  |  | * | * | 6 |
| Cerejeira et al., 2011 | * | * | * | * | * | * | * |  |  | 7 |
| Cerejeira et al., 2012 | * | * | * | * | * | * | * |  |  | 7 |
| Chan et al., 2021 | * |  | * | * |  |  | * | * |  | 5 |
| Chen et al., 2020 | * | * | * | * | * | * | * |  |  | 7 |
| Chen et al., 2019 | * | * | * | * | * | * |  | * |  | 7 |
| Chu et al., 2016 | * | * | * | * | * | * | * | * | * | 9 |
| Cinar et al., 2014 | * | * | * | * | * | * |  | * |  | 7 |
| Conti et al., 2020 | * | * | * | * | * | * |  | * | * | 8 |
| Cooper et al., 2020 | * |  | * |  |  |  |  |  |  | 2 |
| Cunningham et al., 2018 | * | * | * |  | * | * | * |  |  | 6 |
| Dejonghe et al., 2012 | * | * | * |  | * | * | * | * | * | 8 |
| Dillon et al., 2016 | * | * | * | * | * | * | * | * | * | 9 |
| Egberts et al., 2015 | * | * | * |  | * | * | * |  |  | 6 |
| Egberts et al., 2017 | * | * |  |  | * | * |  |  |  | 4 |
| Egberts et al., 2019 | * |  | * |  | * | * | * | * |  | 6 |
| Ehler, Petzold et al., 2019 | * |  | * |  |  |  |  |  |  | 2 |
| Ehler, Saller et al., 2019 | * |  | * |  |  |  |  |  |  | 2 |
| Elsamadicy et al., 2018 | * |  |  |  |  |  |  |  |  | 1 |
| Fernandez et al., 2018 | * |  |  |  |  |  |  |  |  | 1 |
| Fong et al., 2021 | * |  | * |  | * | * | * | * | * | 7 |
| Ford et al., 2013 | * |  | * |  |  |  |  |  |  | 2 |
| Forget et al., 2021 |  |  |  |  |  |  |  |  |  |  |
| Gailiusas et al., 2019 | * | * | * |  | * | * |  |  |  | 5 |
| Girard et al., 2012 | * |  | * |  |  |  |  | * | * | 4 |
| Hall et al., 2013 | * | * | * | * | * | * |  | * | * | 8 |
| Henjum et al., 2018 | * | * | * | * | * | * | * | * |  | 8 |
| Hirsch et al., 2016 | * | * | * | * | * | * |  | * | * | 8 |
| Hov et al., 2017 | * | * | * | * | * | * | * | * |  | 8 |
| Ida et al., 2020 | * | * |  |  | * | * |  |  |  | 4 |
| Idland et al., 2017 | * | * | * | * | * | * | * | * |  | 8 |
| Inoue et al., 2017 | * |  | * |  | * | * | * | * | * | 7 |
| Kalyoncuoglu et al., 2020 | * | * |  |  | * | * |  |  | * | 5 |
| Katsumi et al., 2020 | * | * | * | * | * | * |  | * | * | 8 |
| Kazmierski et al., 2021 | * | * | * | * | * | * |  | * |  | 7 |
| Kazmierski et al., 2013 | * | * | * | * | * | * |  | * |  | 7 |
| Knaak 2019 et al., 2019 | * | * | * |  | * | * |  | * |  | 6 |
| Kotfis, Olejnik et al., 2019 | * | * | * |  | * | * |  | * | * | 7 |
| Kotfis, Slozowska et al., 2019 | * | * | * | * | * | * | * | * |  | 8 |
| Kozak et al., 2016 | * | * | * |  | * | * |  | * | * | 7 |
| Li et al., 2017 | * | * | * | * | * | * |  | * | * | 8 |
| Lin et al., 2020 | * | * | * |  | * | * |  | * |  | 6 |
| Lu et al., 2020 | * | * | * |  | * | * |  | * |  | 6 |
| Ma et al., 2020 | * | * | * | * | * | * | * | * |  | 8 |
| McNeil et al., 2019 | * | * | * | * | * | * |  | * |  | 7 |
| Menzenbach et al., 2021 | * |  | * |  | * | * |  | * |  | 5 |
| Miao et al., 2016 | * | * | * |  | * | * |  | * |  | 6 |
| Michels et al., 2021 | * |  | * | * |  |  |  | * |  | 4 |
| Mietani et al., 2019 | * |  | * |  | * | * |  | * |  | 5 |
| Mossanen et al., 2020 | * |  | * |  |  |  |  |  |  | 2 |
| Neerland et al., 2016 | * | * | * | * | * | * | * | * |  | 8 |
| Nguyen et al., 2014 | * | * | * |  |  |  |  | * | * | 5 |
| Nguyen et al., 2016 | * | * | * |  |  |  | * | * | * | 6 |
| Osse et al., 2012 | * | * | * | * | * | * |  | * | * | 8 |
| Parker et al., 2021 | * | * | * | * | * | * |  | * |  | 7 |
| Peng et al., 2019 | * | * | * |  | * | * | * | * |  | 7 |
| Pfister et al., 2008 | * | * | * |  | * | * |  | * | * | 7 |
| Pilling et al., 2020 | * |  | * | * |  |  |  |  |  | 3 |
| Plaschke et al., 2010 | * | * | * |  | * |  |  |  |  | 4 |
| Plaschke et al., 2016 | * | * | * |  | * | * |  | * | * | 7 |
| Pol et al., 2014 | * | * | * |  | * | * | * |  |  | 6 |
| Rasmussen et al., 2000 | * |  | * |  |  |  |  | * |  | 3 |
| Reznik et al., 2021 | * | * | * |  | * | * |  |  |  | 5 |
| Ritchie et al., 2014 | * | * | * |  | * | * |  | * |  | 6 |
| Sajjad et al., 2020 | * | * | * | * | * | * |  | * | * | 8 |
| Saller et al., 2019 | * | * | * |  | * | * | * | * | * | 8 |
| Saller et al., 2020 | * | * |  |  | * | * | * |  |  | 5 |
| Sanchez et al., 2013 | * | * | * |  | * | * |  |  |  | 5 |
| Sasajima et al., 2000 | * | * | * |  | * | * |  | * | * | 7 |
| Shen et al., 2016 | * | * | * | * | * | * | * | * |  | 8 |
| Shen et al., 2020 | * | * | * |  | * | * |  | * |  | 6 |
| Simons et al., 2018 | * | * | * | * | * | * |  | * | * | 8 |
| Sugita et al., 2018 | * | * |  | * | * | * |  | * | * | 7 |
| Thisayakorn et al., 2021 | * | * | * | * | * | * |  | * |  | 7 |
| Thomas et al., 2008 | * | * | * | * | * |  |  |  |  | 5 |
| Van Munster, Korevaar et al., 2009 | * | * | * | * | * | * | * | * | * | 9 |
| Van Munster, Korse et al., 2009 | * | * | * | * | * | * | * | * | * | 9 |
| Van Munster et al., 2010 | * | * | * | * | * | * | * | * | * | 9 |
| Van Munster et al., 2012 | * | * | * | * | * | * | * | * | * | 9 |
| Wyrobek et al., 2017 | * | * | * | * | * | * |  | * |  | 7 |
| Zhao et al., 2018 | * | * | * |  | * | * |  | * | * | 7 |

#1. Representativeness of patients with delirium

#2. Selection of the comparative patients without delirium

#3. Ascertainment of delirium station

#4. Demonstration that delirium was present or not at the beginning of the study

#5. Study controls for age

#6. Study controls for gender/type of setting/type of surgery

#7. Independent or blind assessment stated in the paper, or confirmation of delirium by reference to secure records

#8. Was follow-up long enough for delirium to occur

#9. Adequacy of follow up of delirium

1. **For evaluation of case-control studies**

| **Author** | **Selection** | | | | **Comparability** | | **Exposure** | | | **Total** |
| --- | --- | --- | --- | --- | --- | --- | --- | --- | --- | --- |
| First author, year | #1 | #2 | #3 | #4 | #5 | #6 | #7 | #8 | #9 |  |
| Fong et al., 2020 | * | * | * | * | * | * | * | * |  | 8 |
| Halaas et al., 2019 | * | * | * | * | * | * | * | * |  | 8 |
| Halaas et al., 2021 | * | * | * | * | * | * | * | * |  | 8 |
| Han, Zhang et al., 2020 | * | * | * | * | * | * | * | * |  | 8 |
| Han, Chen et al., 2020 | * | * | * | * | * | * | * | * |  | 8 |
| Kalantar et al., 2018 | * | * | * | * | * | * | * | * |  | 8 |
| Neerland et al., 2020 | * | * | * | * | * | * | * | * |  | 8 |
| Pan et al., 2019 | * | * | * | * | * | * | * | * |  | 8 |
| Poljak et al., 2014 | * | * | * | * | * | * | * | * |  | 8 |
| Rudolph et al., 2008 | * | * | * | * | * | * | * | * |  | 8 |
| Saito et al., 2020 | * | * | * | * | * | * | * | * |  | 8 |
| Sun et al., 2016 | * | * | * | * | * | * | * | * |  | 8 |
| Szwed et al., 2020 | * | * | * | * | * | * | * | * |  | 8 |
| Szwed et al., 2021 | * | * |  | * |  |  | * | * |  | 6 |
| Van den Boogaard, Kox et al., 2011 | * | * | * | * | * | * | * | * |  | 8 |
| Van den Boogaard, van Swelm et al., 2011 | * | * | * | * | * | * | * | * |  | 8 |
| Van Munster et al., 2011 | * | * | * | * | * | * | * | * |  | 8 |
| Vasunilashorn et al., 2018 | * | * | * | * | * | * | * | * |  | 8 |
| Vasunilashorn et al., 2021 | * | * | * | * | * | * | * | * |  | 8 |
| Wu, Xu et al., 2017 | * | * | * | * | * | * | * | * |  | 8 |
| Yuan et al., 2020 | * | * | * | * | * | * | * | * |  | 8 |

#1. Is delirium definition adequate?

#2. Representativeness of patients with delirium

#3. Selection of the comparative patients without delirium

#4. Definition of controls

#5. Study controls for age

#6. Study controls for gender/type of setting…

#7. Ascertainment of case-control station

#8. Same method of ascertainment of case-control station

#9. Same non-response rate for cases and controls

MoCA=Montreal Cognitive Assessment; TMT-B=Trail Making Test part B; GFI=Groningen Frailty Indicator; HDS-R=Hasegawa´s dementia scale;
