# Supplementary material for: Biomarkers of delirium risk in older adults: a systematic review and meta-analysis
Source: Front Aging Neurosci. 2023 May 12;15:1174644. doi: 10.3389/fnagi.2023.1174644 (PMC10213257; doi:10.3389/fnagi.2023.1174644)
Supplement: Supplementary file 3 [file Data_Sheet_3.DOCX]

Supplementary Material


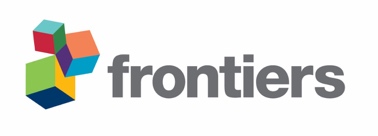
Biomarkers of delirium risk in older adults:

a systematic review and meta-analysis

Lozano-Vicario, Lucía^1*^; García-Hermoso, Antonio^2^; Cedeño-Veloz, Bernardo Abel^1^; Fernández-Irigoyen, Joaquín^3^; Santamaría, Enrique^3^; Romero-Ortuno, Román^4^; Zambom-Ferraresi, Fabricio^2,5^; L. Sáez de Asteasu, Mikel^2^; Muñoz-Vázquez, Ángel Javier^6^; Izquierdo, Mikel^2^; Martínez-Velilla, Nicolás^1,2,5^

*** Correspondence:**

Corresponding Author: Lozano-Vicario, Lucía lucia.lozanovicario@gmail.com

***Supplementary data 3****.* ***Baseline characteristics of included studies***

**a. Neurotransmitters**

| **Authors (year)**  **Provenance** | **Design**  **Setting** | **Biomarker(s)**  **Source of biomarker**  **Method used** | **Timing of sampling** | **Sample size**  **Cases**  **Controls** | **Age (years)**  **Mean or median**  **Sex (% male)** | **Delirium assessment tool**  **Delirium severity**  **Delirium subtype** | **Main results** |
| --- | --- | --- | --- | --- | --- | --- | --- |
| Adam et al.  (2020)  Germany | Prospective cohort study  Elective cardiac surgery | AChE  BChE  Blood  ChE Check Mobile | Preoperatively and on the on the 1^st^ and 2^nd^ postoperative day | N=114  Delirious=31  Non-delirious=83 | Delirious=74 (71%)  Non-delirious=69 (73.5%) | CAM-ICU preoperatively and on the 1^st^ and 2^nd^ postoperative days  Not reported  Not reported | -↓preoperative AChE activity in delirious group  -No association between BChE and POD |
| Cerejeira et al. (2012)  Portugal | Prospective cohort study  Elective hip surgery | AChE  BChE  Blood  xMAP Bio-Plex Suspension Array System 200 | Preoperatively and on the 1^st^ postoperative day | N=101  Delirious=37  Non-delirious=64 | Delirious=73.6 (40.5%)  Non-delirious=72.6 (54.6%) | CAM in the evening of the surgery day and was repeated on 2^nd^ and 3^rd^ postoperative days  Not reported  Not reported | -In delirious patients preoperative levels of plasma cholinesterase activity correlated with CRP, IL -6, proinflammatory/anti-inflammatory ratio |
| De Jonghe et al. (2012)  The Netherlands | Prospective cohort study  Hip fracture | Tryptophan, kynurenine and IDO activity  Blood  Fluorescence using an isocratic, reversed phase HPLC system | Samples were taken during weekdays whenever patients were available for venipuncture | N=140  Delirious=71  Non-delirious=69 | Delirious=85.1 (24%)  Non-delirious=82.5 (36%) | CAM daily  Not reported  Not reported | -↑ preoperative kynurenine/tryptophan ratio is a risk marker for POD  -↑ IDO activity was related with incident delirium |
| Ma et al. (2020)  China | Prospective cohort study  PCI in acute coronary syndrome | Ach  Blood  ELISA | Before PCI | N=119  Delirious=51  Non-delirious=100 | Delirious=75.7 (68.4%)  Non-delirious=67.7 (74%) | DSM criteria preoperative and within 7 days after PCI  Not reported  Not reported | -↓ Ach level is an independent risk factor for delirium |
| Osse et al. (2012)  The Netherlands | Prospective cohort study  Elective cardiac surgery | Amino acids (HVA)  Blood  HPLC | The day before surgery and the 2^nd^ postoperative day | N=125  Delirious=58  Non-delirious=67 | Delirious=76.7 (71.6%)  Non-delirious=75.1 (58.6%) | CAM-ICU before surgery and daily after surgery until discharge  Not reported  Not reported | -↑ postoperative HVA levels were associated with POD |

**b. Hormones**

| **Authors (year)**  **Provenance** | **Design**  **Setting** | **Biomarker(s)**  **Source of biomarker**  **Method used** | | **Timing of sampling** | **Sample size**  **Cases**  **Controls** | | **Age (years)**  **Mean or median**  **Sex (% male)** | **Delirium assessment tool**  **Delirium severity**  **Delirium subtype** | **Main results** |
| --- | --- | --- | --- | --- | --- | --- | --- | --- | --- |
| Avila-Funes et al. (2015)  Mexico | Prospective cohort study  Medical inpatients | Estradiol and cortisol  Blood  Estradiol-radioimmunoassay (COAT-A-COUNT kit)  Cortisol-radioimmunoassay (IMMULITE analyzer) | | The morning after admission | N=141  Delirious=23  Non-delirious=118 | | Delirious=79.4 (0%)  Non-delirious=77.5 (0%) | CAM daily until discharge  Not reported  Not reported | -↑ estradiol levels at admission had an increased risk for delirium  -No differences were found between cortisol and delirium |
| Li et al.  (2017)  China | Prospective cohort study  Critically ill patients | Leptin  Blood  ELISA | | The day after ICU admission | N=336  Delirious=102  Non-delirious=234 | | Delirious= 67.9 (72.5%)  Non-delirious= 55.9 (70.9%) | CAM-ICU twice daily maximum of 28 days or until death  Not reported  Not reported | -↓ leptin at ICU entry was independently associated with delirium |
| Ma et al. (2020)  China | Prospective cohort study  PCI in acute coronary syndrome | | Cortisol  Blood  ELISA | Before PCI | N=119  Delirious=51  Non-delirious=100 | Delirious=75.7 (68.4%)  Non-delirious=67.7 (74%) | | DSM criteria preoperative and within 7 days after PCI  Not reported  Not reported | -↑ cortisol is an independent risk factor for delirium |

**c. Neuronal damage**

| **Authors (year)**  **Provenance** | **Design**  **Setting** | | **Biomarker(s)**  **Source of biomarker**  **Method used** | **Timing of sampling** | | **Sample size**  **Cases**  **Controls** | | **Age (years)**  **Mean or median**  **Sex (% male)** | **Delirium assessment tool (when)**  **Delirium severity**  **Delirium subtype** | **Main results** |
| --- | --- | --- | --- | --- | --- | --- | --- | --- | --- | --- |
| Fong et al. (2020)  USA | | Subanalysis of SAGES study: Case-control study  Major elective surgery | NfL and UCHL-1  Blood  Simoa | At 3 moments: preoperative, 2^nd^ day after surgery and 1 month after surgery | | N=108  Delirious=54  Non-delirious=54 | | Delirious=77 (47%)  Non-delirious=77 (47%) | CAM daily  CAM-S  Not reported | -↑ preoperative NfL levels were associated with POD and severity of POD  -No association was found between delirium and UCHL-1 |
| Halaas et al.  (2021)  Norway | | Case-control study  Hip fracture surgery | Neurogranin  CSF  ELISA | | -CSF was collected at the time of anesthesia in surgery patients  -CSF was collected in outpatients | Hip fracture N=128  Delirium=70  Non- delirious=58  Elective surgery cohort N=127  AD patients cohort N=46 | Hip fracture  Delirious patients=85 (30%)  Non-delirious patients=84 (22%)  Elective surgery cohort  72 (50%)  AD patients cohort 67 (39%) | | CAM daily until the 5^th^ postoperative day (all) or until discharge (delirium)  MDAS  Delirium subtype | -No association was found between delirium and CSF neurogranin concentration |
| Hov et al. (2017)  Norway | | Prospective cohort study  Hip fracture surgery and Elective major surgery | S100B  CSF and blood  Electro-chemiluminescence immunoassay | | CSF was collected at the time of spinal anesthesia  Blood was collected preoperatively and once postoperatively | Elective major surgery without delirium: N=50  Hip fracture:  Non-delirious=46  Prevalent delirium=36  Incident delirium=16 | Hip fracture:  Non-delirious=84 (22%)  Prevalent delirium=85 (25%)  Incident delirium=88 (44%) | | CAM daily until the 5^th^ postoperative day (all) or until discharge (delirium)  MDAS  Not reported | -↑preoperative CSF S100B correlated with POD who also had pathological levels of the AD biomarker p-tau |
| Saller et al. (2019)  Germany | | Prospective cohort study  Elective cardiac surgery | NfL  Blood  Simoa method | | Before surgery and after surgery: in ICU, the 1^st^ postoperative day, the 3^rd^ postoperative day and before discharge | N=9  Delirious=3  Non-delirious=6 | 74.9 (88.9%) | | CAM-ICU 3 times per day by nurses and additionally twice per day by the study team and completed with a chart review  Not reported  Not reported | -No differences were found between preoperative NfL and risk of POD |
| Szwed et al. (2020)  Poland | | Case-control study  Elective cardiac surgery | pNfLH  Blood  ELISA | | At baseline (before surgery), at the end of surgery, and postoperative days 1 and 7 | N=100  Delirious=26  Non-delirious=74 | Delirious=67.2 (73.1%)  Non-delirious=64 (72.9%) | | CAM-ICU 24h after surgery twice daily until the 7^th^ postoperative day  Not reported  Delirium subtype with RASS scale | No differences were found between NfL and POD |

**d. Neuroinflammation**

| **Authors (year)**  **Provenance** | **Design**  **Setting** | | **Biomarker(s)**  **Source of biomarker**  **Method used** | **Timing of sampling** | | | **Sample size**  **Cases**  **Controls** | **Age (years)**  **Mean or median**  **Sex (% male)** | **Delirium assessment tool**  **Delirium severity**  **Delirium subtype** | **Main results** | |
| --- | --- | --- | --- | --- | --- | --- | --- | --- | --- | --- | --- |
| Cape et al. (2014)  Scotland | Prospective cohort study  Hip fracture surgery | | IL-1β, IL-1ra, IFN-γ, IGF-I, GFAP  CSF and blood  ELISA | Before administration spinal anesthesia | | | N=43  Delirious=19  Non-delirious=24 | Delirious=81.3 (26%)  Non-delirious=81.3 (25%) | CAM pre- operatively on the day of surgery, followed by a postoperative assessment 3–4 days later | -↑CSF IL-1β in patients with incident delirium  -No differences were found between the rest of biomarkers and POD | |
| Cerejeira et al. (2012)  Portugal | Prospective cohort study  Elective hip surgery | | CRP, IL-1β, TNF-α, IL-6, IL-8, IL-10  Blood  xMAP Bio-Plex Suspension Array System 200 | Preoperatively and on the 1^st^ postoperative day | | | N=101  Delirious=37  Non-delirious=64 | Delirious=73.6 (40.5%)  Non-delirious=72.6 (54.6%) | CAM in the evening of the surgery day and was repeated on 2^nd^ and 3^rd^ postoperative days  Not reported  Not reported | -↑preoperative CRP in patients who developed delirium  -No differences were found between the rest of biomarkers and incident delirium | |
| Chen et al. (2020)  China | Prospective cohort study  Elective gastric cancer surgery | | CRP, IL-6, TNF-α  Blood  ELISA | The morning before surgery | | | N=370  Delirious=63  Non-delirious=307 | Delirious=70.9 (68.2%)  Controls=70.4 (62.8%) | DSM-V criteria at 1 day before surgery and 1-7 days (once daily in the evening) after surgery  Not reported  Not reported | -↑CRP was a risk factor for POD  -No differences were found between the rest of biomarkers and incident delirium | |
| Chen et al. (2019)  China | Prospective cohort study  Elective cardiac surgery | | IL-6  Blood  ELISA | Before the induction of anesthesia on the morning of surgery and at the 6th, 12th, and 18th postoperative hours | | | N=266  Delirious=85  Non-delirious=181 | Delirious=67 (56%)  Non-delirious= 61 (82%) | CAM-ICU before surgery and twice daily postoperatively from day 1 to day 5  Not reported  Not reported | -↑IL-6 is a predictor of POD | |
| Chu et al. (2016)  Taiwan | Prospective cohort study  Orthopedic elective surgery | | IGF-I  Blood  ELISA | The morning before surgery | | | N=103  Delirious=23  Non-delirious=80 | Delirious=81.1 (78.3%)  Non-delirious=81.9 (72.5%) | CAM daily until discharge  Not reported  Not reported | -No association was found between preoperative IGF-I levels and POD | |
| Çinar et al. (2014)  Turkey | Prospective cohort study  Elective cardiac surgery | | CRP, IGF-I, TNF-α  Blood  ELISA | Before surgery and on the 2^nd^ postoperative day | | | N=35  Delirious =15  Non-delirious=20 | Delirious =69 (46.6%)  Non-delirious=60.7 (95%) | DRS daily and DSM-IV criteria, from postoperative day 2 to day 7  DRS  Not reported | -↓ preoperative IGF-1 were associated with development of POD  -Preoperative CRP and TNF-α levels were not different between groups. | |
| Dillon et al. (2016)  Israel | Prospective cohort study  Major elective surgery | | CRP  Blood  ELISA | At 4 time points: preoperative, post-anesthesia care unit, postoperative day 2, and 1 month postoperative | | | Discovery cohort:  N=78  Delirious=39  Non-delirious=39  Replication cohort:  N=72  Delirious =36  Non-delirious=36  Pooled cohort:  N=150  Delirious =75  Non-delirious=75 | Discovery cohort:  Delirious=77.3 (46%)  Non-delirious=76.8 (46%)  Replication cohort:  Delirious=78 (42%)  Non-delirious=77.6 (42%)  Pooled cohort:  Delirious =77.6 (44%)  Non-delirious=77.2 (44%) | CAM daily  Not reported  Not reported | -↑preoperative CRP were associated with POD | |
| Fong et al. (2020)  USA | Subanalysis of SAGES study: Case-control study  Major elective surgery | | GFAP, UCHL-1  Blood  Simoa | At 3 moments: preoperative, 2^nd^ day after surgery and 1 month after surgery | | | N=108  Delirious =54  Non-delirious=54 | Delirious =77 (47%)  Non-delirious=77 (47%) | CAM daily  CAM-S  Not reported | -No association was found between GFAP, UCHL-1 and POD or delirium severity | |
| Hirsch et al. (2016)  USA | Prospective cohort study  Orthopedic elective surgery | | IFN-γ, IFN-α2, IL-10, IL-12p70, IL-2, IL-4, IL-5, IL-6, IL-8, TNF-α, MCP-1, MIP-1α, MIP-1β, RAGE, Calprotectin MRP8/14  Blood and CSF  Milliplex kit and ELISA | CSF: at the time of anesthesia  Blood: preoperatively and 3,6 and 18h postoperatively | | | N=10  Delirious =1  Non-delirious=9 | 70.3 (70%) | CAM pre and postoperatively daily until hospital discharge  Not reported  Not reported | - ↑Plasma calprotectin, MIP-1α, MIP-1β and IL-6 at baseline in patients who developed POD.  -↑ CSF IL-6, IL-8 and calprotectin in patients who developed POD.  -↓Preoperative plasma IFN-α2, IL-4, IL-5, IFN-γ and IL-12 in patients who developed POD.  -↓ CSF IFN-α2 and RAGE in patients who developed POD  -No differences were found between the rest of biomarkers and incident delirium | |
| Katsumi et al. (2020)  USA | Prospective cohort study  Major orthopedic surgery | | CRP, IL-6 and CHI3L1  CSF and blood  ELISA | Preoperatively and 1 month postoperatively | | | N=36  Delirious =6  Non-delirious=30 | Delirious =74.7 (17%)  Non-delirious=74.3 (17%) | CAM daily  CAM-S  Not reported | -No significant relationships were identified between these biomarkers and delirium | |
| Kazmierski et al. (2021)  Poland | Prospective cohort study  Cardiac elective surgery | | hsCRP and MCP-1  Blood  ELISA | The day prior to surgery and the 1^st^ day postoperatively | | | N=177  Delirious =61  Non-delirious=116 | Delirious=70 (61%)  Non-delirious=66 (87%) | CAM-ICU once a day until the 5^th^ day after surgery  Not reported  Not reported | - ↑preoperative hsCRP was associated with incident delirium  -↑ MCP-1 before surgery was independently associated with POD | |
| Osse et al. (2012)  The Netherlands | | Prospective cohort study  Elective cardiac surgery | Neopterin  Blood  HPLC | | The day before surgery and the 2^nd^ postoperative day | N=125  Delirious=58  Non-delirious=67 | | Delirious=76.7 (71.64%)  Non-delirious=75.1 (58.62%) | CAM-ICU before surgery and daily after surgery until discharge  Not reported  Not reported | | -↑preoperative neopterin was associated with POD |
| Peng et al. (2019)  China | | Prospective cohort study  Elective orthopedic surgery | TNF-α, CAR, IL-6  Blood  ELISA | | Preoperatively | N=272  Delirious=55  Non-delirious=217 | | Delirious=74.5 (40%)  Non-delirious=72.1 (42.9%) | DSM V criteria daily within postoperative 7 days  Not reported  Not reported | | -↑preoperative CAR was an independent risk factor for POD  - ↑preoperative TNF-α and IL-6 in patients with incident POD but not significative |
| Sajjad et al. (2020)  Norway | | Prospective cohort study  3 cohorts: hip fracture surgery, cognitively healthy patients undergoing elective surgery and patients with AD dementia | TNF-α, IL1ß and IL-8  CSF  Mesoscale Discovery Immunoassay | | Before spinal anesthesia in surgery patients and as part of the diagnostic work up for the AD patients | N=382  Hip fracture=137  -Delirious=67 (22 incident delirium and 45 preoperative delirium)  -Non-delirious=61  Elective surgery=172  AD dementia=73 | | Hip fracture=84 (27%)  Elective surgery=71 (50%)  AD=66 (42%) | CAM preoperatively and daily until the 5^th^ postoperative day  Not reported  Not reported | | -↑preoperative IL-8 in patients who developed POD  -TNFα and IL1ß were undetected in most patients |
| Saller et al. (2019)  Germany | | Prospective cohort study  Elective cardiac surgery | GFAP  Blood  ELISA | | Before surgery and after surgery: in ICU, the 1^st^ postoperative day, the 3^rd^ postoperative day and before discharge | N=9  Delirious=3  Non-delirious=6 | | 74.9 (88.9%) | CAM-ICU 3 times per day by nurses and additionally twice per day by the study team and completed with a chart review  Not reported  Not reported | | -No association between GFAP and incident delirium |
| Shen et al. (2016)  China | | Prospective cohort study  Elective gastrointestinal tumor surgery | IGF-I, CRP and IL-6  Blood  ELISA | | The morning on the day before surgery | N=140  Delirious=36  Non-delirious=104 | | Delirious=73.8 (47.2%)  Non-delirious=68.8 (41.3%) | CAM twice daily preoperatively and on the 1^st^ and 2^nd^ postoperative days  DRS-R-98  Not reported | | -↑ preoperative CRP and IL-6 and ↓ IGF-I were associated with higher incidence of POD |
| Szwed et al. (2020)  Poland | | Case-control study  Elective cardiac surgery | GFAP  Blood  ELISA | | At baseline (before surgery), at the end of surgery, and postoperative days 1 and 7 | N=100  Delirious=26  Non-delirious=74 | | Delirious=67.2 (73.1%)  Non-delirious=64 (72.9%) | CAM-ICU 24h after surgery twice daily until the 7^th^ postoperative day  Not reported  Delirium subtype with RASS scale | | No association was found between GFAP and incident POD |

**e. Biomarkers of dementia**

| **Authors (year)**  **Provenance** | **Design**  **Setting** | **Biomarker(s)**  **Source of biomarker**  **Method used** | **Timing of sampling** | **Sample size**  **Cases**  **Controls** | **Age (years)**  **Mean or median**  **Sex (% male)** | **Delirium assessment tool**  **Delirium severity**  **Delirium subtype** | **Main results** |
| --- | --- | --- | --- | --- | --- | --- | --- |
| Fong et al. (2020)  USA | Subanalysis of SAGES study: Case-control study  Major elective surgery | t-tau  Blood  Simoa | At 3 moments: preoperative, 2^nd^ day after surgery and 1 month after surgery | N=108  Delirious=54  Non-delirious=54 | Delirious=77 (47%)  Non-delirious=77 (47%) | CAM daily  CAM-S  Not reported | -No association was found between t-tau and POD or delirium severity |
| Hirsch et al. (2016)  USA | Prospective cohort study  Orthopedic elective surgery | Aß40 and Aß42  Blood and CSF  Milliplex kit and ELISA | CSF: at the time of anesthesia  Blood: preoperatively and 3,6 and 18h postoperatively | N=10  Delirious =1  Non-delirious=9 | 70.3 (70%) | CAM pre and postoperatively daily until hospital discharge  Not reported  Not reported | -↓plasma preoperative Aß40 and Aß42 in the patient with incident POD |
| Hov et al. (2017)  Norway | Prospective cohort study  Hip fracture surgery and Elective major surgery | p-tau  CSF and blood  Electro-chemiluminescence immunoassay | CSF was collected at the time of spinal anesthesia  Blood was collected preoperatively and once postoperatively | Elective major surgery without delirium: N=50  Hip fracture:  Non-delirious=46  Prevalent delirium=36  Incident delirium=16 | Hip fracture:  Non-delirious=84 (22%)  Prevalent delirium=85 (25%)  Incident delirium=88 (44%) | CAM daily until the 5^th^ day postoperative (all) or until discharge (delirium)  MDAS  Not reported | -↑CSF p-tau correlated with ↑CSF S100B and incident POD |
| Pan et al. (2019)  UK | Case-control study  Elective orthopedic surgery | Aß-42, t-tau and p-tau  CSF (blood was also collected)  ELISA | CSF was collected prior to spinal anesthesia  Blood was collected preoperatively | N=54  Delirious=28  Non-delirious=26 | Delirious=76.2 (72%)  Non-delirious=75.9 (74%) | CAM once daily postoperatively, for the first 3 days after surgery  Not reported  Not reported | -↓CSF Aß-42 predicts incident POD  -No association was found between tau and incident delirium |
| Saller et al. (2019)  Germany | Prospective cohort study  Elective cardiac surgery | Tau  Blood  ELISA | Before surgery and after surgery: in ICU, the 1^st^ postoperative day, the 3^rd^ postoperative day and before discharge | N=9  Delirious=3  Non-delirious=6 | 74,9 (88.9%) | CAM-ICU 3 times per day by nurses and additionally twice per day by the study team and completed with a chart review  Not reported  Not reported | -↑preoperative tau in patients with later delirium |

**f. Genetics**

| **Authors (year)**  **Provenance** | **Design**  **Setting** | **Biomarker(s)**  **Source of biomarker**  **Method used** | **Timing of sampling** | **Sample size**  **Cases**  **Controls** | **Age (years)**  **Mean or median**  **Sex (% male)** | **Delirium assessment tool**  **Delirium severity**  **Delirium subtype** | **Main results** |
| --- | --- | --- | --- | --- | --- | --- | --- |
| Chen et al. (2020)  China | Prospective cohort study  Elective gastric cancer surgery | miR-210  Blood  nCode miRNA First Strand cDNA Synthesis kit (Invitrogen) | The morning before surgery | N=370  Delirious=63  Non-delirious=307 | Delirious=70.9 (68.2%)  Non-delirious=70.4 (62.8%) | DSM-V criteria at 1 day before surgery and 1-7 days (once daily in the evening) after surgery  Not reported  Not reported | -↑preoperative expression of miR-210 was a predictor for POD |

**g. Metabolomics, lipidomics and proteomics**

| **Authors (year)**  **Provenance** | **Design**  **Setting** | **Biomarker(s)**  **Source of biomarker**  **Method used** | **Timing of sampling** | **Sample size**  **Cases**  **Controls** | **Age (years)**  **Mean or median**  **Sex (% male)** | **Delirium assessment tool**  **Delirium severity**  **Delirium subtype** | **Main results** |
| --- | --- | --- | --- | --- | --- | --- | --- |
| Han, Zhang et al. (2020)  China | Case-control study  Hip fracture surgery | Metabolomics and lipidomics  CSF  Ultimate 3000 UHPLC system | In the moment of spinal anesthesia (before surgery) | N=40  Delirious =10  Non-delirious=30 | Delirious= 82.2 (50%)  Non-delirious= 81.7 (23.3%) | CAM-Chinese revision twice daily on the 1^st^ and 2^nd^ days after surgery  MDAS  Not reported | -↓preoperative PE (40:7e) was associated with incident delirium  -↓preoperative PE (40:6), PE (38:7e), PE (40:7e), PC (40:6), and PC (33:1) in patients with incident POD but preoperative Cer-NS and SM classes were ↑ |
| Han, Chen et al. (2020)  China | Case-control study  Hip fracture surgery | Proteomics  CSF  LC-MS/MS Analyses | In the moment of spinal anesthesia (before surgery) | N=40  Delirious =10  Non-delirious=30 | Delirious=82.2 (50%)  Non-delirious=81.7 (23.3%) | CAM-Chinese revision twice daily on the 1^st^ and 2^nd^ days after surgery  MDAS  Not reported | - ↓preoperative VSTM2B and FA5 were positively correlated with delirium severity |
| Pan et al. (2019)  UK | Case-control study  Elective orthopedic surgery | Spermidine, glutamine and putrescine  CSF (blood was also collected)  Quantitative mass spectrometry-based metabolomic | CSF was collected prior to spinal anesthesia  Blood was collected preoperatively | N=54  Delirious=28  Non-delirious=26 | Delirious=  76.2 (72%)  Non-delirious=  75.9 (74%) | CAM once daily postoperatively, for the first 3 days after surgery  Not reported  Not reported | ↑Spermidine, glutamine and putrescine in patients who developed delirium |
| Vasunilashorn et al. (2018)  USA | Case-control study  Major non-cardiac surgery | Proteomics (CRP, AZGP1, SERPINA 3)  Blood  Mass spectrometry proteomics and ELISA | 1 sample before surgery and another sample the 2^nd^ postoperative day | N=560  Delirious=134  Non-delirious=426  (12 delirious and 12 non-delirious pairs were selected for this study) | 77 (44%) | CAM daily  Not reported  Not reported | - ↓ preoperative AZGP1 and ↑CRP were associated with incident POD |
| Vasunilashorn et al. (2021)  USA | Case-control study  Major non-cardiac surgery | Proteomics  Blood  SOMAscan and ELISA | 1 sample before surgery and another sample the 2^nd^ postoperative day | N=36  Delirious=18  Non-delirious=18 | 76 (43%) | CAM daily  CAM-S  Not reported | -↑preoperative CHI3L1/YKL-40 was associated with incident POD |

**h. Others**

| **Authors (year)**  **Provenance** | | **Design**  **Setting** | **Biomarker(s)**  **Source of biomarker**  **Method used** | **Timing of sampling** | | **Sample size**  **Cases**  **Controls** | **Age (years)**  **Mean or median**  **Sex (% male)** | **Delirium assessment tool**  **Delirium severity**  **Delirium subtype** | **Outcome** |
| --- | --- | --- | --- | --- | --- | --- | --- | --- | --- |
| Bakker et al. (2012)  The Netherlands | | Prospective cohort study  Elective cardiac surgery | Creatinine  Blood  Not reported | Before surgery | | N=201  Delirious =63  Non-delirious=138 | Delirious=76.7 (59%)  Non-delirious=75.9 (61%) | CAM-ICU daily until 7 days after surgery  Not reported  Not reported | -↑ creatinine prior to surgery is an independent predictor of POD |
| Kotfis, Slozowska et al. (2019)  Poland | | Retrospective analysis of a prospective cohort study  Cardiac elective surgery | WDC and platelets  Blood  Not reported | Preoperatively and on the 1^st^, 3^rd^ and 5^th^ postoperative days | | N=968  Delirious=129  Non-delirious=839 | Delirious=71.69 (67.44%)  Non-delirious=65.37 (77.95%) | CAM-ICU in the cardiac-ICU and twice daily during the first 6 days after surgery during hospitalization  Not reported  Not reported | -↓ preoperative levels of PLR and PWR were associated with incident POD |
| Szwed et al. (2020)  Poland | Case-control study  Elective cardiac surgery | | NSP and VILIP-1  Blood  ELISA | | At baseline (before surgery), at the end of surgery, and postoperative days 1 and 7 | N=100  Delirious=26  Non-delirious=74 | Delirious=67.2 (73.1%)  Non-delirious=64 (72.9%) | CAM-ICU 24h after surgery twice daily until the 7^th^ postoperative day  Not reported  Delirium subtype with RASS scale | -↑ end of surgery to baseline ratio of neuroserpin predicted the occurrence of POD.  -No differences were found between VILIP-1 and delirium |
| Wyrobek et al. (2017)  USA | Prospective cohort study  Spine surgery | | BDNF  Blood  Electrochemiluminescent sandwich immunoassay | | Preoperatively and hourly during surgery | N=77  Delirious=32  Non-delirious=45 | 75 (53%) | CAM and CAM-ICU daily during the first 4 days after surgery  DRS-R-98  Not reported | -↓intraoperative BDNF was associated with incident POD |

**Abbreviations**

CAM-ICU= confusion assessment method for the intensive care unit; CAM= Confusion Assessment Method; DRS= Delirium Rating Scale; CAM-S=CAM Severity; POD=postoperative delirium; HPLC=high-performance liquid chromatography; UHPLC=Ultra High Performance Liquid Chromatograph; ELISA=enzyme-linked immunosorbent assay; CSF=cerebrospinal fluid; AD=Alzheimer Disease; Simoa= single-molecule array enzyme linked immunoassay; SAGES study=The Successful Aging after Elective Surgery; LC-MS/MS=Liquid Chromatography Tandem-Mass Spectrometry; PCI=percutaneous coronary intervention

↓=low levels; ↑=high levels
